# 12‐HETE is an Endogenous Modulator of BLT2 Triggering Vascular Degeneration, Dissection, and Rupture

**DOI:** 10.1002/advs.202515897

**Published:** 2025-11-05

**Authors:** Yuyu Li, Jiaqi Yu, Weiyao Chen, Xue Wang, Xin Tan, Jing Cui, Yihao Wang, Xuan Xu, Rui Lin, Zhengkai Wang, Wenxi Jiang, Yongliang Zhong, Jie Du, Yuan Wang

**Affiliations:** ^1^ Beijing Anzhen Hospital Capital Medical University Key Laboratory of Remodeling‐related Cardiovascular Diseases Ministry of Education Beijing Collaborative Innovation Centre for Cardiovascular Disorders No. 2 Anzhen Road, Chaoyang District Beijing 100029 China; ^2^ Beijing Institute of Heart Lung and Blood Vessel Disease No. 2 Anzhen Road, Chaoyang District Beijing 100029 China; ^3^ Department of Cardiology The First Affiliated Hospital of Wenzhou Medical University Wenzhou Medical University Wenzhou Zhejiang 325015 China; ^4^ Department of Cardiology The Second Affiliated Hospital of Wenzhou Medical University Wenzhou Medical University Wenzhou Zhejiang 325027 China; ^5^ Department of Nutrition The First Affiliated Hospital of Sun Yat‐sen University Guangzhou Guangdong 510080 China

**Keywords:** aortic dissection, inflammation, macrophage, smooth muscle cells

## Abstract

Thoracic aortic dissection (TAD), a life‐threatening vascular emergency requiring urgent pharmacological intervention, prompted investigation into arachidonic acid metabolites based on their established roles in inflammation and vascular homeostasis. Through comprehensive plasma metabolomics analysis performed in both TAD patients and a β‐aminopropionitrile monofumarate (BAPN)‐induced mouse model, significant elevation of 12‐hydroxyeicosatetraenoic acid (12‐HETE) was identified, accompanied by marked upregulation of its synthetic enzyme 12/15‐lipoxygenase (12/15‐LOX) in dissected aortic tissues. The pathogenetic significance was further demonstrated by the substantial protection against TAD progression that was observed in Alox15‐deficient mice. Mechanistically, 12‐HETE was confirmed to function as an endogenous ligand for the BLT2 receptor on macrophages, leading to activation of the NOX‐1/ROS/NF‐κB signaling cascade. This signaling activation was shown to induce inflammatory cytokine release and promote inflammatory cell recruitment, ultimately resulting in pathological phenotype switching of vascular smooth muscle cells. The therapeutic potential was validated by significant reduction in dissection rupture rates that was achieved with either the 12/15‐LOX inhibitor ML351 or the BLT2 antagonist LY255283, with maximal efficacy being demonstrated when both agents were administered in combination. These findings establish the 12‐HETE‐BLT2 axis as a key driver of TAD pathogenesis and validate its potential as a promising therapeutic target for clinical intervention.

## Graphical abstract

In thoracic aortic dissection (TAD), elevated levels of the arachidonic acid metabolite 12‐HETE, produced by macrophage‐specific 12/15‐lipoxygenase (12/15‐LOX), bind to the BLT2 receptor on vascular cells. This activates the NOX‐1/ROS/NF‐κB signaling pathway, leading to IL‐6 release. IL‐6 promotes an inflammatory cascade and, via the JAK/STAT pathway, induces a contractile‐to‐synthetic phenotypic switch in vascular smooth muscle cells (VSMCs), resulting in extracellular matrix degradation, aortic wall weakening, and dissection. Genetic or pharmacological inhibition of 12/15‐LOX (with ML351) or BLT2 (with LY255283) blocks this pathway and mitigates TAD.

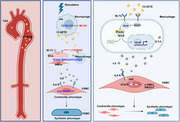

## 1 Introduction

Thoracic aortic dissection (TAD) is a severe medical condition with rapid progression and high morbidity in the acute phase.^[^ ^1^, ^2^ ^]^ Although surgical therapies have significantly improved in recent years, there are insufficient clinically effective medications to slow or prevent aortic degeneration.^[^ ^3^ ^]^ Therefore, investigations of TAD pathogenesis are needed to develop effective treatment strategies.

TAD pathogenesis is complex and involves inflammation, apoptosis, smooth muscle cell phenotype switching, and extracellular matrix degradation.^[^ ^4^, ^5^, ^6^ ^]^ Vascular smooth muscle cells (VSMCs) comprise the majority of cellular components in the aorta, and their phenotypes range from a quiescent (contractile) state (responsible for regulating blood flow and pressure) to a proliferative (synthetic) phenotype (capable of synthesizing matrix metalloproteinase MMP2 and MMP9).^[^ ^7^ ^]^ Aberrant VSMC phenotype switching is triggered by environmental stimuli such as inflammatory cytokines and reactive oxygen species (ROS).^[^ ^8^ ^]^ In response to inflammatory cues, VSMCs undergo dedifferentiation to a synthetic phenotype, which exhibits enhanced proliferative and migratory capacities.

Arachidonic acid metabolism is a potent regulator of inflammation.^[^ ^9^ ^]^ Arachidonic acid, typically distributed in the cell membrane in phospholipid form, is released in response to pathological stimuli. The released arachidonic acid is converted into eicosanoids (prostaglandins and leukotrienes), thromboxanes, and other signaling lipid metabolites that may trigger inflammatory responses.^[^ ^10^, ^11^ ^]^ Bioactive metabolites of arachidonic acid can also trigger cardiomyocyte ferroptosis, thereby promoting myocardial ischemia‐reperfusion injury.^[^ ^12^, ^13^ ^]^ There is experimental evidence that arachidonic acid and its metabolites are involved in pathological processes including oxidative stress, cardiomyocyte apoptosis, and platelet hyperactivation.^[^ ^14^, ^15^ ^]^ Thus, they play important roles in cardiovascular diseases such as myocardial infarction and hypertension. However, the potential contributions of these eicosanoids to TAD development have not been investigated. We speculated that the coordinated release of arachidonic acid metabolites after vascular injury initiates an inflammatory cascade and subsequent VSMC phenotype switching, which may be an important contributing factor in TAD formation.

Here, we performed an arachidonic acid‐targeted metabolomics analysis using plasma samples collected from TAD patients and healthy controls. We observed substantial accumulation of LOX family‐associated metabolites in TAD plasma, including 12‐hydroxyeicosatetraenoic acid (12‐HETE), a product of arachidonate 12/15‐lipoxygenase (12/15‐LOX). We found that 12/15‐LOX was increased in diseased aortic tissue from TAD patients and TAD mice. Our observations in an animal model validated this highly conserved role for 12/15‐LOX in TAD. Furthermore, we found that 12‐HETE, detected in early vascular injury, induces aortic inflammation by activating BLT2 and promoting aberrant VSMC phenotype switching. Overall, we identified 12‐HETE as a key initiator of the inflammatory response that promotes VSMC phenotype switching during TAD formation, then demonstrated its utility as a therapeutic target for TAD prevention.

## 2 Results

### 2.1 12/15‐LOX Expression and Plasma 12‐HETE Levels Were Elevated in TAD Patients and TAD Mice

**Figure 1:**
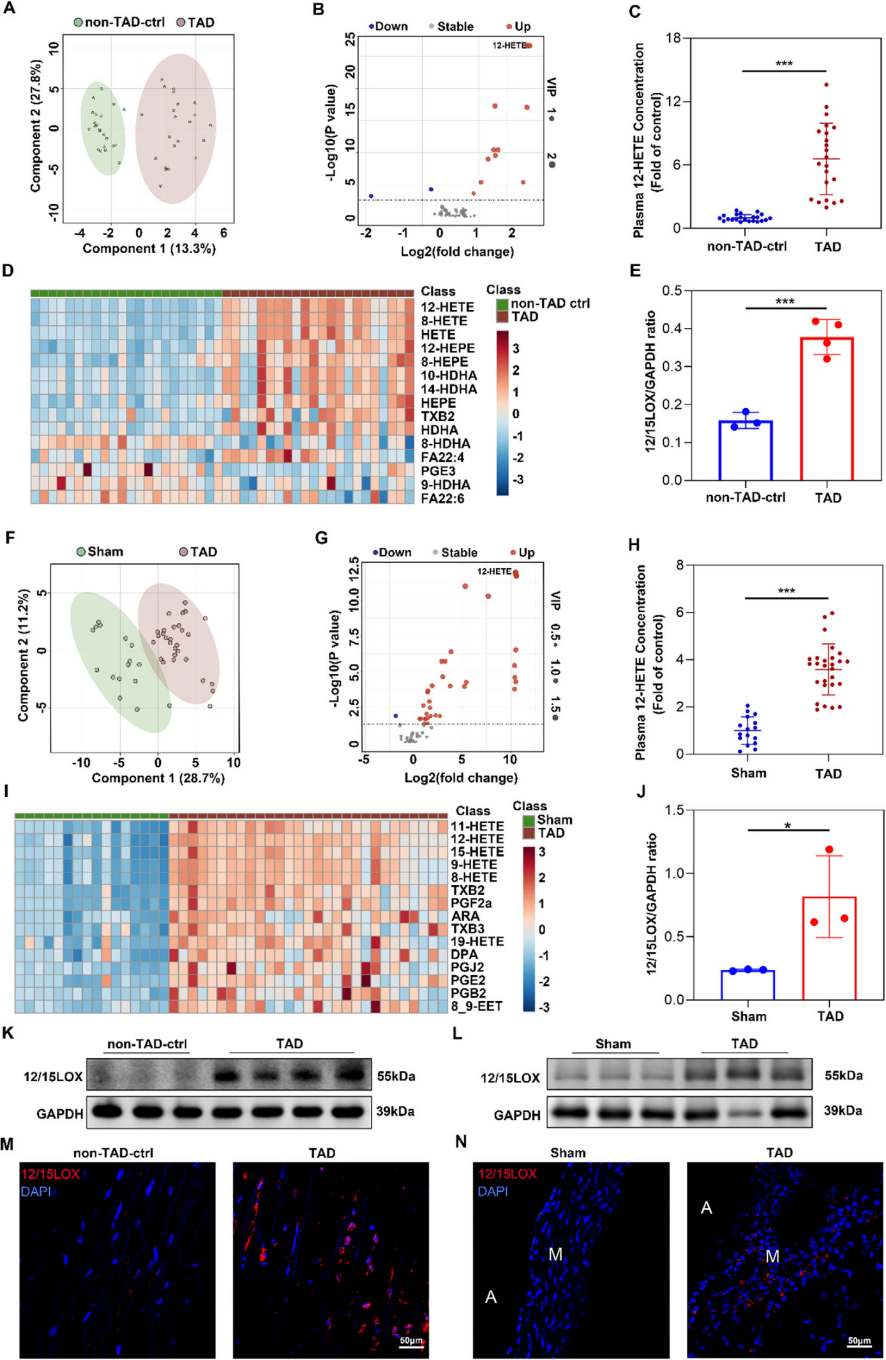
Metabolomics profiling identifies Alox15‐12‐HETE as the most outstanding axis in TAD. A) AA metabolites were extracted and interpreted by Partial Least Squares Discriminant Analysis (PLS‐DA), and the 2D score plots display repertoires of non‐TAD control and TAD patients. Each point represents a sample, and ellipses represent 95% confidence regions (*n* = 22 and 22). B) Volcano plot shows − log10(P‐value) on the *y*‐axis versus log2(fold change) on the *x*‐axis. Each point represents a different metabolite, and the greater the scattered point, the greater the value of variable importance is in the projection (VIP). C) Plasma 12‐HETE concentration (fold of control) of non‐TAD controls and TAD individuals (*n* = 39 and 47). D) Plasma contents of AA pathway metabolites of TAD individuals and non‐TAD controls based on targeted metabolomics. E) Western blot analysis and quantification of 12/15‐LOX expressed in the aorta of controls without TAD and patients with TAD (*n* = 3 and 4 per group). F) AA metabolites were extracted and interpreted by PLS‐DA, and the 2D score plots display repertoires of healthy control and BAPN‐induced TAD model mice. Each point represents a sample, and ellipses represent 95% confidence regions (*n* = 16 and 27). G) Volcano plot of AA metabolites from the plasma of healthy control and TAD mice. H) Plasma 12‐HETE concentration of healthy control and TAD mice (*n* = 16 and 27). I) Plasma contents of AA pathway metabolites of healthy control and TAD mice based on targeted metabolomics. J) Western blot analysis and quantification of 12/15‐LOX expressed in healthy control and TAD mice (*n* = 3 per group). K) Western blot analysis of 12/15‐LOX levels in the aorta of controls without TAD and patients with TAD (*n* = 3 and 4 per group). L) Western blot analysis of 12/15‐LOX levels in healthy control and TAD mice (*n* = 3 per group). M) Representative images of human aorta stained with 12/15‐LOX (12/15‐LOX; red) and DAPI (blue). Scale bars = 50 µm; media (M). N) Representative images of mouse aorta stained with 12/15‐LOX (12/15‐LOX; red) and DAPI (blue). Scale bars = 50 µm; media (M); adventitia. ^*^ *p* < 0.05, ^**^ *p* < 0.01 and ^***^ *p* < 0.001. Data were presented as the mean ± SD and analyzed by using an unpaired two‐tailed Student's *t* test.

To identify metabolites potentially associated with TAD, we performed arachidonic acid‐targeted metabolomics analyses of plasma samples from 22 TAD patients and 22 age‐sex‐matched healthy controls in a discovery cohort (Figure S1A, Supporting Information). In total, 74 metabolites were detected including 4 polyunsaturated fatty acids (ARA, EPA, DPA, DHA) and 70 eicosanoids across the cyclooxygenase (COX), lipoxygenase (LOX), and cytochrome P450 (CYP450) pathways. Furthermore, partial least square discriminant analysis (PLS‐DA), a mathematical procedure that decreases data dimensionality while preserving most of the variance by orthogonal transformation,^[^ ^16^ ^]^ indicated that AA metabolite variables were distinguishable between TAD patients and healthy controls (**Figure** **1**A). Heatmap analysis of the levels of 74 metabolites revealed considerable differences between TAD patients and healthy controls (Figure 1D). Among these AA metabolites, 12‐HETE exhibited the greatest alteration and was identified as the main contributor to the overall difference in plasma samples from human participants (Figure 1B; Figure S1B, Supporting Information). Additionally, relative quantification of 12‐HETE by mass spectrometry showed that plasma concentrations were significantly greater in TAD patients than in healthy controls (Figure 1C).

In the single‐center validation cohort, we measured the plasma level of 12‐HETE in 218 TAD and 138 healthy controls for further validation. The baseline table of single‐center validation was presented in Table S2 (Supporting Information). Elevated plasma 12‐HETE levels in TAD patients (median: 11.89 ng mL^−1^, IQR: 6.22–23.59) were found, compared with healthy controls (median: 3.35 ng mL^−1^, IQR: 2.28–4.87), as shown in Figure S1F (Supporting Information). Plasma concentration of 12‐HETE remained an independent predictor of aortic dissection, after adjusting for common risk factors (including age, gender, smoking and drinking habits, history of hypertension and diabetes, and systolic blood pressure), as shown in Figure S1G (Supporting Information). Besides, we evaluated the predictive ability of 12‐HETE using the area under the receiver operating characteristic curve. The discriminant performance of the baseline model (AUROC = 0.86, 95%CI: 0.92‐0.96, reference) was further improved by the addition of 12‐HETE (AUROC = 0.95, 95%CI: 0.82–0.90, *p*< 0.001), as shown in Figure S1H (Supporting Information). Therefore, plasma 12‐HETE levels had a positive diagnostic performance of patients with TAD from healthy controls.

Furthermore, we analyzed 27 plasma samples from β‐aminopropionitrile monofumarate (BAPN)‐treated TAD mice and 16 plasma samples from sham mice (Figure S1A, Supporting Information). PLS‐DA indicated that AA metabolites were distinguishable between BAPN‐treated TAD and sham mice (Figure 1F,G). 12‐HETE exhibited the greatest alteration in TAD mice, compared to sham mice (Figure 1H,I; Figure S1C, Supporting Information).

The 12‐HETE was implicated as a primary metabolite in the 12/15‐LOX pathway.^[^ ^17^ ^]^ 12/15‐LOX, encoded by Alox15 is an enzyme that can produce 12‐HETE from arachidonic acid. Using western blotting (WB) and immunofluorescence (IF) staining analysis, we showed that 12/15‐LOX expression in aortic tissues was significantly greater among TAD patients than among healthy controls (Figure 1K,M; Figure S1D, Supporting Information). Increased expression of 12/15‐LOX was also observed in BAPN‐treated TAD mice (Figure 1L,N; Figure S1E, Supporting Information). WB quantification of 12/15‐LOX is depicted in Figure 1E,J. These results suggest that 12/15‐LOX and its metabolite 12‐HETE have roles in TAD development.

### 2.2 Global Alox15 Knockout Mitigates TAD Development in Mice

**Figure 2:**
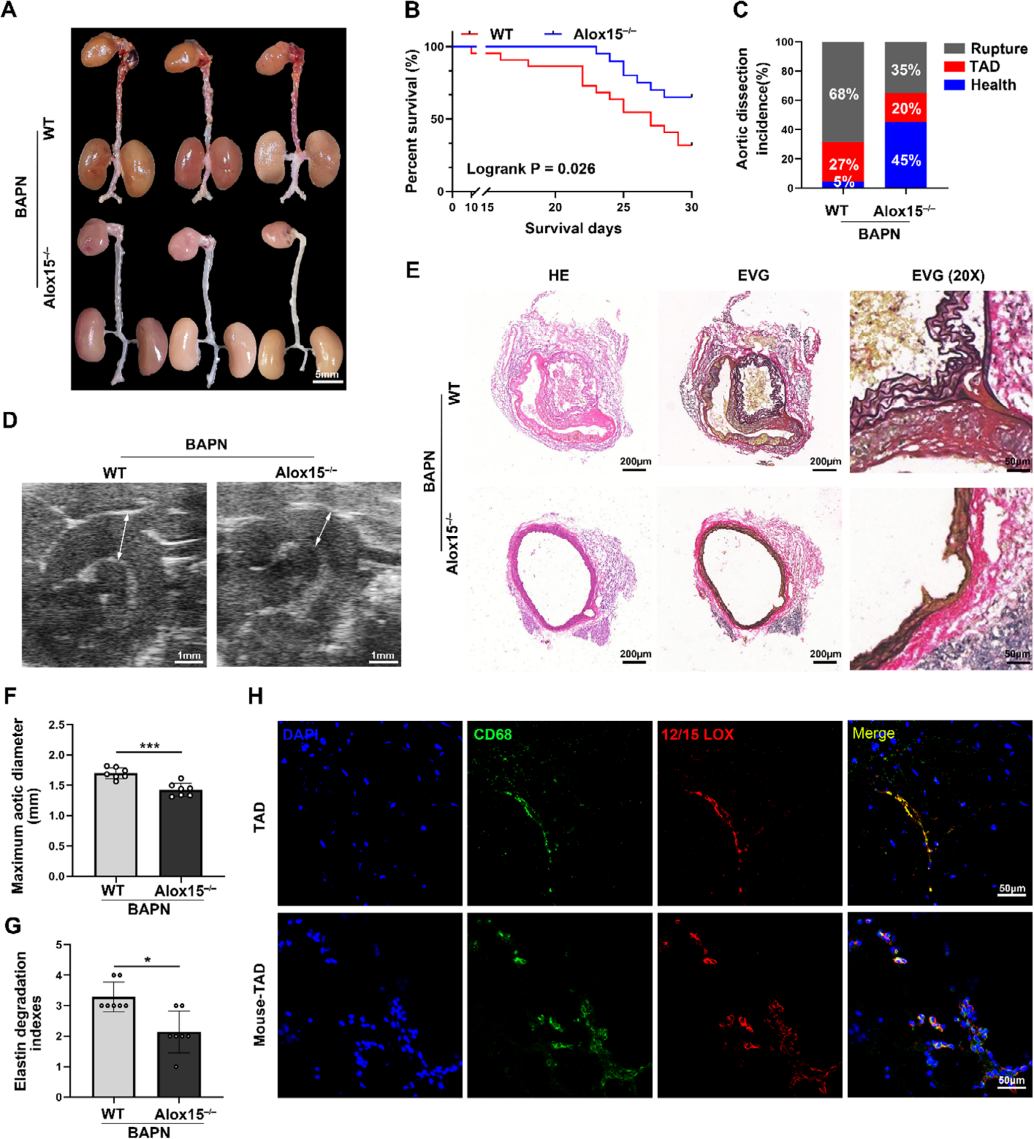
Global Alox15 knockout mitigates TAD development in mice. A‐F, Wild‐type (WT) and Alox15^−/−^ mice were treated with BAPN (β‐aminopropionitrile monofumarate) for 28 days. A) Representative macrographs of the aorta (scale bar = 5 mm). B) Survival rate was estimated by Kaplan–Meier method and compared by the log‐rank test (P = *n* = 22 and 20). C) Thoracic aortic dissection (TAD) incidence. D) Representative ultrasound images of thoracic aorta. E) Representative macroscopic images of aorta sections stained with hematoxylin and eosin (HE) and Elastic Van Gieson (EVG) (scale bars, 200 and 50 µm). F) Measurements of maximum aortic diameter (*n* = 7 per group). G) Elastin break grades were analyzed by Kruskal–Wallis followed by Dunn multiple comparisons test (*n* = 7 per group). H) Representative confocal images of 12/15‐LOX (red) colocalized with CD68 (green)–positive macrophages in aorta tissues from patients with thoracic aortic dissection (TAD) and TAD mice (scale bars = 50 µm). ^*^ *p* < 0.05 and ^***^ *p* < 0.001. Data were presented as the mean ± SD and analyzed by using an unpaired two‐tailed Student's *t* test.

To investigate the role of 12‐HETE in aortic dissection, we performed in vivo experiments using a mouse model of BAPN‐induced TAD. First, global 12/15‐LOX knockout mice (Alox15^−/−^) were purchased from the Jackson Laboratory. Next, 4‐week‐old male 12/15‐LOX knockout mice (*n* = 20) and C57BL/6J wild‐type (WT) mice (*n* = 22) were treated with BAPN for 28 days. During the 28 days of BAPN administration, 68% (*n* = 15) of the C57BL/6J WT mice and 35% (*n* = 7) of the Alox15^–/–^ mice died of aortic dissection and rupture (**Figure** **2**B,C). 27% and 20% of total WT and Alox15 knockout mice developed TAD (Figure 2A,C). Vascular ultrasound images and measurements of maximum aortic diameter on day 28 after modeling also demonstrated that Alox15 knockout mitigated BAPN‐induced aortic dilation compared with WT controls (Figure 2D,F). Hematoxylin and eosin (HE) staining and Elastica van Gieson (EVG) staining showed that dissecting aneurysm formation and elastin disruption were also alleviated in BAPN‐treated Alox15^−/−^ mice compared with C57BL/6J mice (Figure 2E,G). In the absence of BAPN treatment, WT and Alox15^−/−^ mice exhibited minimal differences in aortic wall thickness, diameter, and TAD formation (Figures S2A–C, Supporting Information). Body weight (Figure S2D, Supporting Information), systolic pressure (Figure S2E, Supporting Information), and diastolic pressure (Figure S2F, Supporting Information) showed minimal differences between Alox15^−^ mice and WT controls. In addition, 4‐week‐old female 12/15‐LOX knockout mice (*n* = 20) and C57BL/6J wild‐type (WT) mice (*n* = 20) were treated with BAPN for 28 days. As expected, with 12/15‐LOX knockout, the TAD progression was also improved in female mice (Figure S3, Supporting Information). In addition, 12/15‐LOX was dramatically elevated in aorta of patients with TAD and TAD mice, primarily located in macrophages (Figure 2H), in accordance with previous reports. These findings suggested that 12/15‐LOX deficiency can reduce TAD formation.

### 2.3 Alox15 Deficiency Abolishes Injury‐Induced Contractile‐to‐Synthetic Phenotype Switch in VSMCs

**Figure 3:**
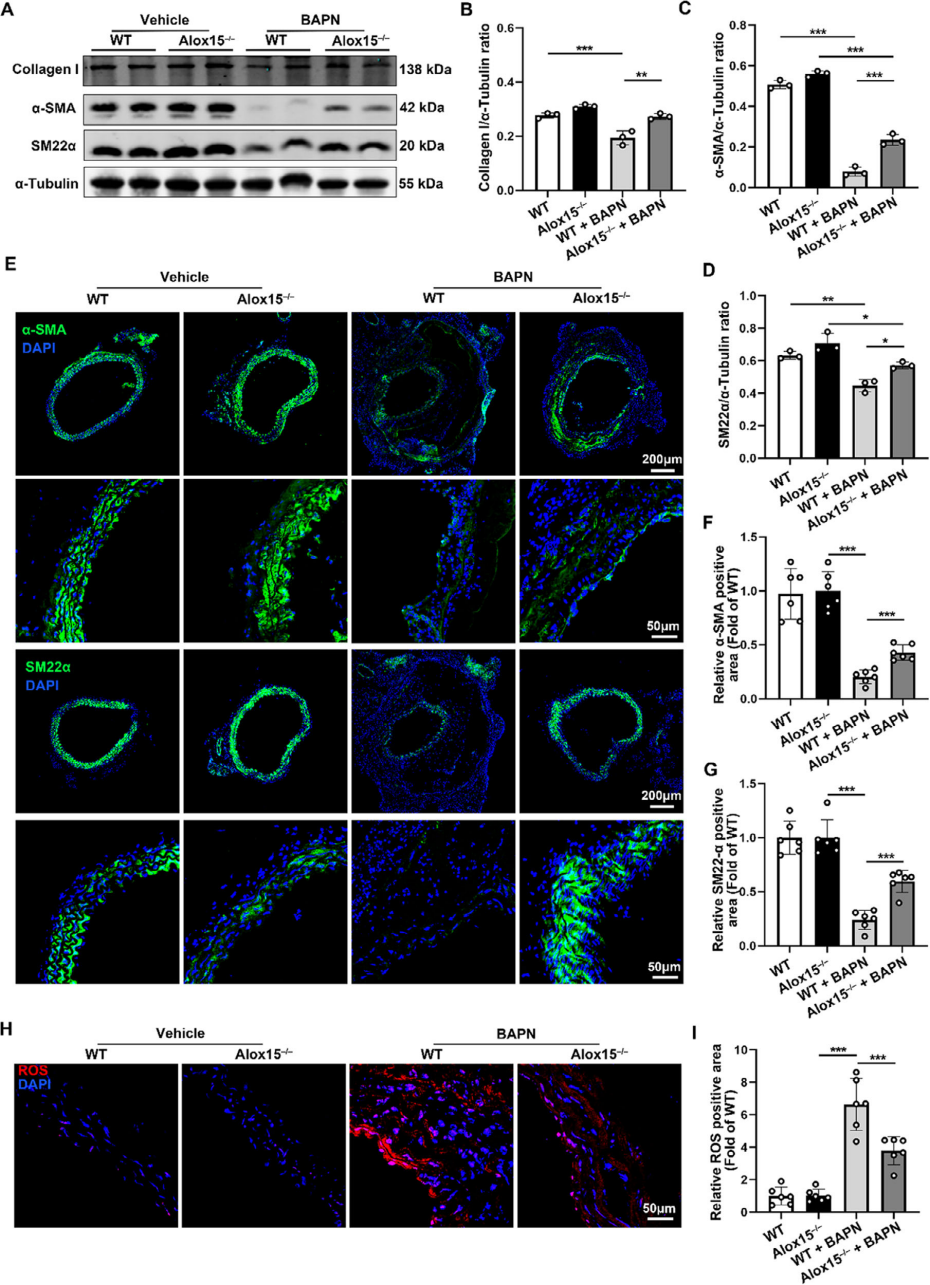
Alox15 deficiency abolishes injury‐induced contractile‐to‐synthetic phenotype switch in VSMCs A–I, Wild‐type (WT) and Alox15^−/−^ mice were treated with or without BAPN for 28 days. A) Representative Western blot images of Collagen I, α‐SMA, and SM22α in TAD aorta. B–D) Western blot analysis and quantification of Collagen I, α‐SMA, and SM22α expression in the aorta of BAPN – and saline‐treated WT and Alox15^−/−^ mice (*n* = 3 per group). E) Immunofluorescence staining of α‐SMA (Green) and SM22α (Green) in aorta. Nuclei were counterstained with DAPI (blue; scale bars, 200 µm, 50 µm). F,G) Quantification of α‐SMA and SM22α– positive area in aorta (*n* = 6 per group). H,I) The level of reactive oxygen species (ROS) in the aorta section of BAPN – and saline‐treated WT and Alox15^−/−^ mice were evaluated by dihydroethidium (DHE) staining and quantified by determining the ratio of DHE‐positive area (*n* = 6) (scale bars, 50 µm). ^*^ *p* < 0.05, ^**^ *p* < 0.01, and ^***^ *p* < 0.001, data were presented as the mean ± SD and analyzed by using one‐way ANOVA, Tukey's multiple comparisons test.

Considering that the contractile‐to‐synthetic phenotype switch in VSMCs is a key pathogenic component of TAD development, we measured the expression levels of aorta‐associated proteins such as α‐smooth muscle actin (α‐SMA), smooth muscle 22α (SM22α), and collagen I. WB analysis revealed that Alox15 deletion had minimal effects on basal levels of contractile markers such as SM22α and α‐SMA. After BAPN treatment, WT mice exhibited a significant decrease in contractile markers in the aorta, whereas Alox15 deletion rescued these levels (**Figure** **3**A–D). IF staining demonstrated that Alox15 deletion counteracted undesirable BAPN‐induced changes in SM22α and α‐SMA (Figure 3E–G). Moreover, the protein levels of MMP2 and MMP9, which promote TAD formation, were considerably higher in TAD tissues than in adjacent non‐TAD tissues. Additionally, MMP2 and MMP9 were downregulated in the aorta of Alox15^−/−^ TAD mice, indicating that extracellular matrix formation was inhibited in such mice (Figure S4, Supporting Information). Those results suggested that Alox15 deficiency can activate differentiation signaling in VSMCs and inhibit extracellular matrix degradation.

Considering that ROS are key signaling molecules with important roles in the progression of inflammatory disorders, we examined ROS levels in the aortas of BAPN‐ and saline‐treated mice. As shown in Figure 3H,I, Alox15 deletion alleviated ROS production in the aorta of BAPN‐treated TAD mice. Taken together, these results suggest that Alox15 silencing is associated with decreased formation of BAPN‐induced TAD.

### 2.4 Alox15 Deficiency Reduces BAPN‐Induced Vascular Inflammation in Mice

**Figure 4:**
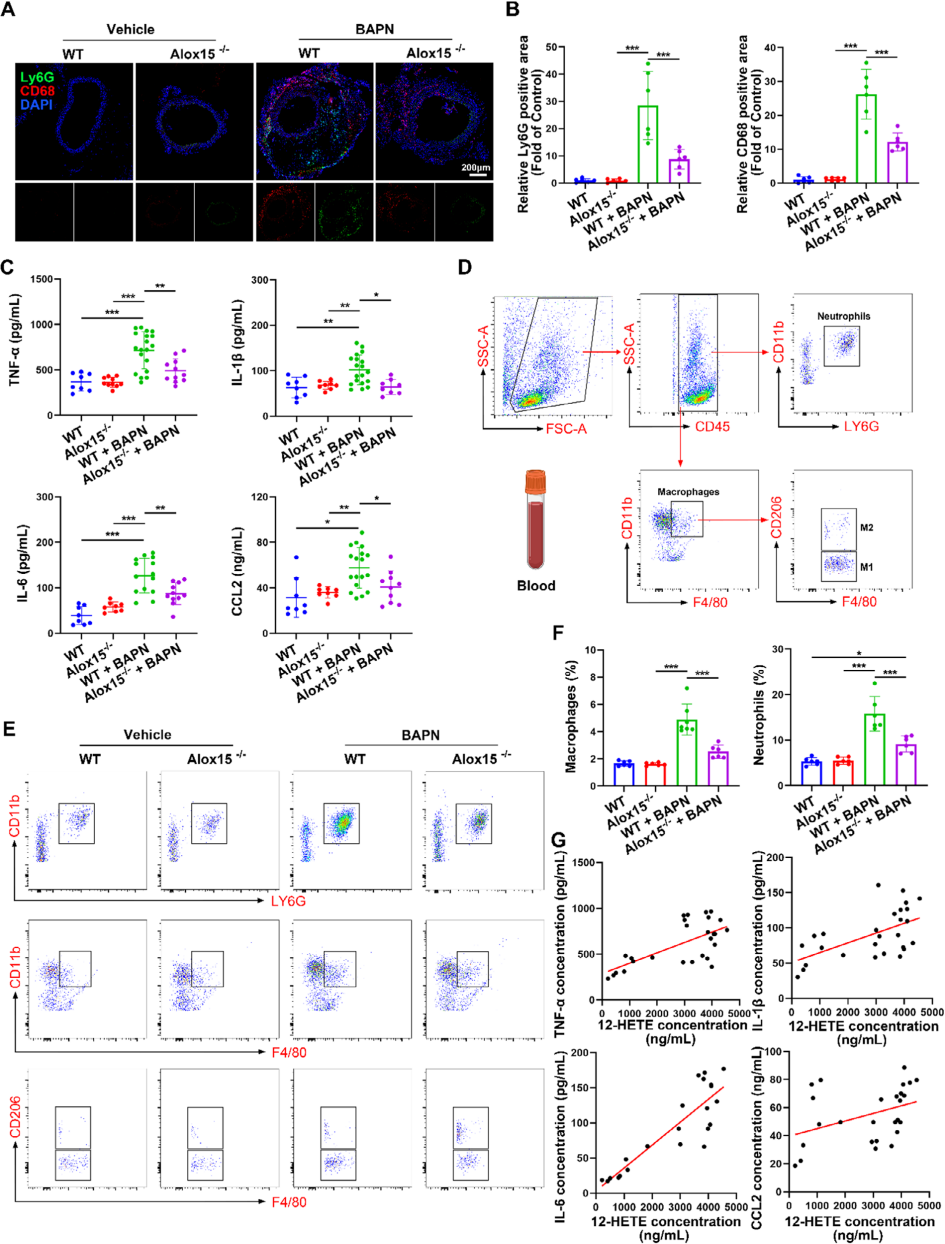
Alox15 deficiency reduces BAPN‐induced vascular inflammation in mice. A) Representative images of frozen aorta sections stained with an antibody against Ly6G (green) and CD68 (red) nuclei were stained with DAPI (blue). Scale bars, 200 µm. B) The statistical graph of the results is shown on the right (*n* = 6 per group). The Ly6G‐positive and CD68‐positive areas were measured and expressed as the percentage of the positive area in sections out of the entire visual field of the section. C) ELISA analysis of inflammatory factors TNF‐α, IL‐1β, IL‐6, and CCL2 in murine plasma at the endpoint. D) Gating strategy for identification of macrophages and neutrophils in mouse blood. Macrophages were identified as CD45+CD11b+ F4/80+ and further classified as CD206+ and CD206‐, neutrophils as CD45+CD11B+LY6G+. E) Representative flow cytometry analysis of circulating blood Ly6G+ CD11b+ neutrophils and CD11b + F4/80+ macrophages in BAPN – and saline‐treated WT and Alox15^−/−^ mice. F) Flow cytometry data analysis was performed as shown. G) Plasma 12‐HETE concentrations are positively correlated with plasma TNF‐α, IL‐1β, IL‐6 and CCL2 levels in mice (*n* = 27, 27, 22, 26 per group). ^*^ *p* < 0.05, ^**^ *p* < 0.01, and ^***^ *p* < 0.001, data were presented as the mean ± SD and analyzed by using one‐way ANOVA, Tukey's multiple comparisons test. Correlation analysis was performed using linear regression and Spearman's method.

Considering that Alox15 deletion can alleviate ROS production in the aorta of TAD mice, we evaluated its effect on the inflammatory response in BAPN‐treated TAD mice. IF staining revealed fewer Ly6G^+^ neutrophils and CD68^+^ macrophages infiltrating the aorta in Alox15^−/−^ TAD mice than in WT TAD mice (**Figure** **4**A,B). Furthermore, the expression levels of inflammatory factors interleukin (IL)‐1β, IL‐6, tumor necrosis factor (TNF)‐α, and CCL2 were decreased in plasma samples from Alox15^−/−^ mice (Figure 4C). It's worth noting that elevated 12‐HETE was observed in mice treated with BAPN for 3 days, precedes the increase in IL‐6, IL‐1β and CCL2 (Figure S5, Supporting Information). Moreover, flow cytometry analysis indicated that there were considerably fewer neutrophils and macrophages in Alox15^−/−^ mice (Figure 4D–F). What's more, Alox15 deletion in myeloid cells resulted in dramatic suppression of myelopoiesis in the bone marrow (BM). There were fewer newly formed neutrophils and macrophages in the BM (Figure S6, Supporting Information), spleen (Figure S7, Supporting Information), and blood (Figure 4E,F). Further, we also performed correlation analyses between plasma 12‐HETE levels and plasma inflammatory factor (IL‐1β, IL‐6, TNF‐α and CCL2) levels, and the results showed that 12‐HETE was positively correlated with these pro‐inflammatory factors (Figure 4G). These findings demonstrated that 12/15‐LOX deficiency had a critical role in regulating TAD inflammation.

### 2.5 Macrophage‐Specific Deletion of Alox15 Protects from BAPN‐Induced TAD

**Figure 5:**
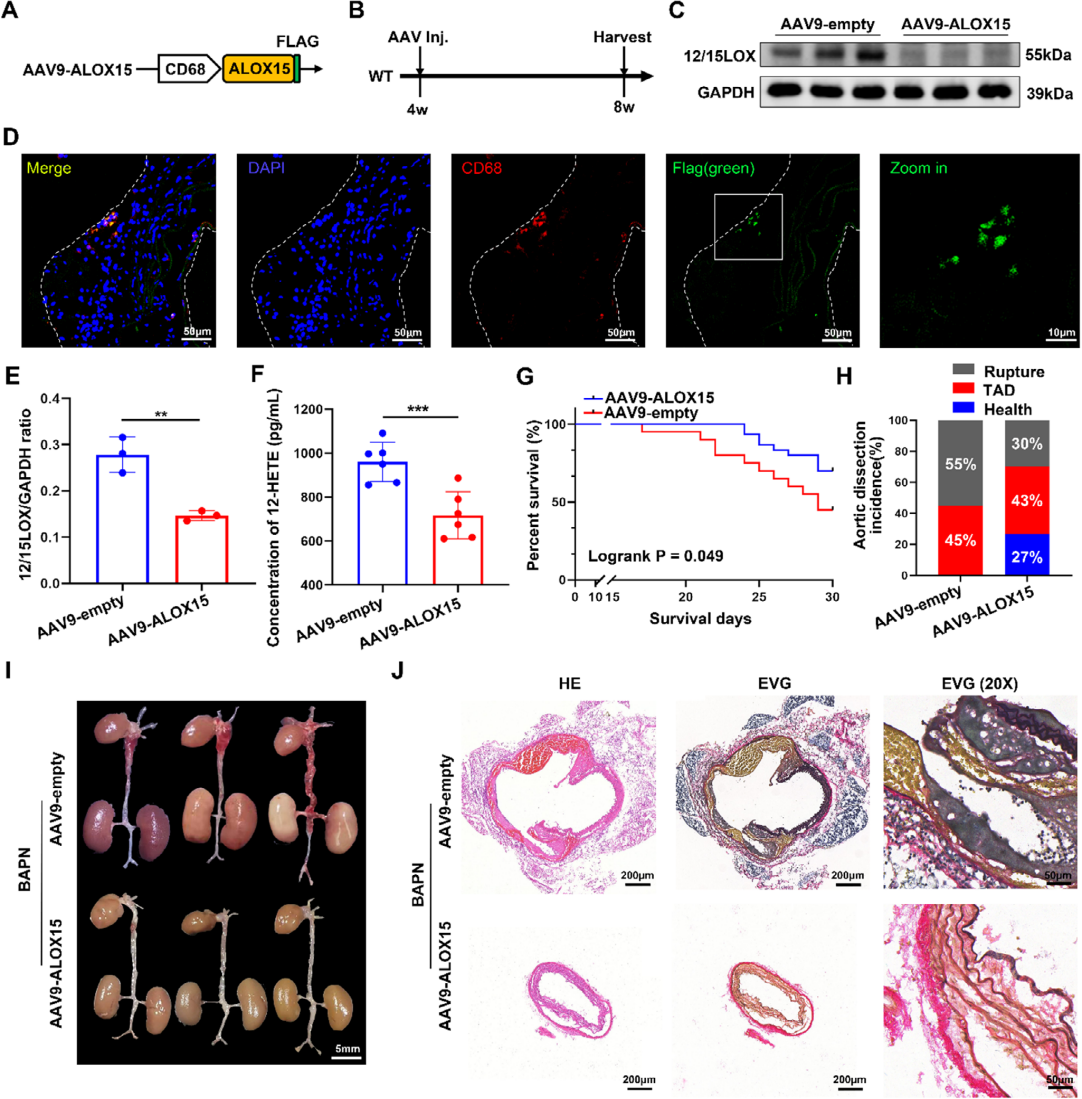
Macrophage‐specific deletion of Alox15 protects from BAPN‐induced TAD. A) Design of an associated adenovirus 9 (AAV9) in which a cDNA coding for Alox15 containing a Flag epitope was expressed under the control of the macrophage‐specific CD68 promoter. B) Experimental scheme of the recombinant AAV9 injected (inj.) into wild type (WT) mice with 1.0 × 10^12^ virus particles at 4 week of age, then harvested at 8 weeks of age. C,E) Western blotting from isolated macrophage from bone marrow of mice previously injected with AAV9‐empty vector or AAV9‐Alox15‐Flag. Glyceraldehyde 3‐phosphate dehydrogenase (GAPDH) Western blotting was used as a control. D) Representative images of BAPN induced TAD aorta stained with CD68 (red) Flag (green) and DAPI (blue). Scale bars, 50 and 10 µm. F) Concentration of plasma 12‐HETE. G, Survival rate was estimated by Kaplan–Meier method and compared by log‐rank test (*n* = 20 and 30). H) Thoracic aortic dissection (TAD) incidence. I) Representative macrographs of aorta (scale bar = 5 mm). J) Representative macroscopic images of aorta sections stained with hematoxylin and eosin (HE) and Elastic Van Gieson (EVG; scale bars, 200 and 50 µm). ^**^ *p* < 0.01, and ***p < 0.001. Data were presented as the mean ± SD and analyzed by using an unpaired two‐tailed Student's *t* test.

To examine the specificity of the Alox15 in causing TAD here, we generated a recombinant AAV9 to delete the 12/15‐LOX protein (Flag tagged) specifically in macrophages using the CD68 promoter (**Figure** **5**A). 4‐week mice were injected with an empty AAV9 vector control or the AAV9‐CD68, followed by analysis at 8 weeks (Figure 5B). Reduced expression of 12/15‐LOX in the macrophages derived from bone marrow after AAV9‐CD68 infection was confirmed by Western blotting versus mice injected with an AAV9‐macrophage‐empty vector (Figure 5C,E). Furthermore, Figure 5D showed that in the aorta of BAPN‐induced TAD mice, macrophages were infected with the AAV9‐CD68 virus (flag tagged). Furthermore, we also measured the plasma 12HETE levels of these two groups of mice after BAPN induction and found that the level in the AAV‐CD68 treatment group had a significant decrease (Figure 5F).

During the 28 days of BAPN administration, 55% (*n* = 11) of the AAV9‐macrophage‐empty vector control mice and 30% (*n* = 9) of the AAV9‐CD68 vector infected mice died of aortic dissection and rupture (Figure 5G,H). HE staining and EVG staining showed that dissecting aneurysm formation and elastin disruption were also alleviated in BAPN‐treated AAV9‐CD68 vector infected mice compared with AAV9‐macrophage‐empty vector control mice (Figure 5I,J).

### 2.6 12‐HETE is an Essential Driver of TAD Development Through the BLT2 Receptor in Mice

Because arachidonic acid can be converted to 12‐HETE through a process mediated by the metabolic enzyme 12/15‐LOX, it has been unclear whether 12‐HETE participates in aortic dissection. We initiated intraperitoneal injection of 12‐HETE in week 2 during BAPN‐induced mouse modeling of TAD. As expected, 12‐HETE injection exacerbated mortality in mice (*n* = 19 and 23, Figure S8B, Supporting Information). Additionally, the rates of aortic dissection formation and rupture increased from 95% (*n* = 18) and 63% (*n* = 12) to 100% (*n* = 23) and 86% (*n* = 20), respectively (Figure S8A,C, Supporting Information). Vascular ultrasound images and measurements of maximum aortic diameter on day 28 after modeling also demonstrated that 12‐HETE exacerbated BAPN‐induced aortic dilation, compared with WT controls Figure S8D,F, Supporting Information). Hematoxylin and eosin staining and Elastica van Gieson staining showed that dissecting aneurysm formation and elastin disruption were worse in 12‐HETE‐treated mice than in saline‐treated mice (Figure S8E,G, Supporting Information). These findings indicated that the metabolite 12‐HETE augmented TAD formation.

BLT2, a receptor for 12‐HETE, is reportedly expressed in endothelial cells (ECs).^[^ ^18^ ^]^ Here, we found that BLT2 was colocalized with the EC marker CD31, as well as the smooth muscle cell marker α‐SMA and the macrophage marker CD68, in the aorta of TAD mice (Figure S9A, Supporting Information). In addition, the results were also confirmed in the aorta of TAD patients (Figure S9B, Supporting Information). Mouse aortic endothelial cells (MAECs), primary smooth muscle cells, and primary macrophages also expressed BLT2 upon stimulation with angiotensin II (Ang II; Figure S10A,B, Supporting Information). Furthermore, 12‐HETE promoted BLT2 expression in primary SMCs and macrophages upon stimulation with Ang II (Figure S10C–E, Supporting Information).

### 2.7 12‐HETE‐Mediated Regulation of VSMC Phenotype Switching Requires IL‐6 Production

**Figure 6:**
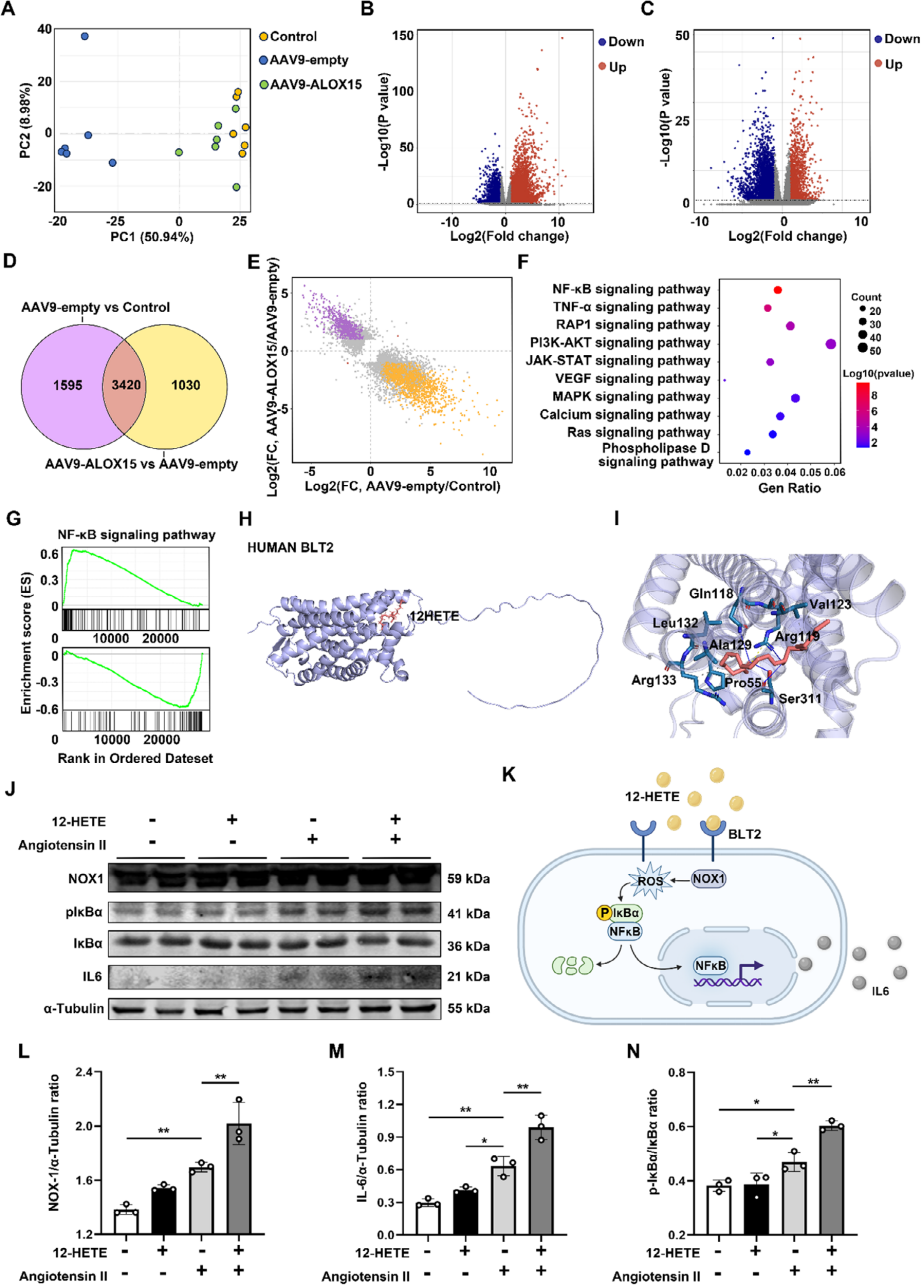
12‐HETE‐mediated regulation of VSMC phenotype switching requires IL‐6 production. A) PCA, and the 2D score plots display repertoires of sham, AAV‐empty TAD mice and AAV‐Alox15 TAD mice. (*n* = 6 each group). B) Volcano plot (AAV‐empty vs sham) shows − log10(P‐value) on the *y*‐axis versus log2(fold change) on the *x*‐axis. Each point represents a different gene. C) Volcano plot (AAV‐Alox15 vs AAV‐empty) shows − log10 (P‐value) on the *y*‐axis versus log2 (fold change) on the *x*‐axis. Each point represents a different gene. D) Venn diagrams of AAV‐empty versus sham and AAV‐Alox15 versus AAV‐empty. E) Log2 FC of common genes in TAD compared to Control (*x*‐axis) and AAV‐Alox15 versus TAD (*y*‐axis). Differential genes with opposite or consistent alteration were marked with different colors. F) Bubble plot shows the enrichment for signal transduction of the KEGG pathway. G) GSEA showing the enrichment score of NFκB signaling pathway in the aorta tissue of AAV‐empty versus sham and AAV‐Alox15 versus AAV‐empty. H,I) Representative docking images of 12‐HETE and BLT2 from human. J) Representative Western blot images of NOX‐1, p‐IκBα, IκBα, and IL‐6 in primary macrophages. K) Graphical abstract. 12‐HETE induced NOX‐1/ROS/NFκb activation via BLT2 receptor. L–N) Western blot analysis and quantification of NOX‐1, IL‐6, and p‐IκBα/IκBα expressed in macrophages (*n* = 3 per group). ^*^ *p* < 0.05, and ^**^ *p* < 0.01. Data were presented as the mean ± SD and analyzed by using one‐way ANOVA, Tukey's multiple comparisons test.

In order to further explore the mechanism of the macrophage‐specific Alox15 in regulating TAD, we performed RNAseq analysis on mouse aorta harvested from sham group (PBS), BAPN‐treated AAV9‐CD68 vector infected mice, and with AAV9‐macrophage‐empty vector control mice (**Figure** **6**A). Compared with the sham group, the AAV9‐empty group (BAPN‐induced TAD) had a total of 5015 differential genes in the aorta of mice (Figure 6B). Gene Ontology analysis revealed that up‐regulated genes associated with the AAV9‐empty group (BAPN‐induced TAD) were significantly enriched in inflammatory response (Figure S11A, Supporting Information). What's more, to reveal the potential mechanism of macrophage‐specific Alox15 in TAD, a comparison between the AAV9‐empty group and the AAV9‐Alox15 group showed 4450 differential genes (Figure 6C). In addition, the GO enrichment in up‐regulated genes in AAV‐Alox15 versus AAV‐empty were also analyzed and the results showed that defense response to other organism was the most significant different pathway (Figure S11A, Supporting Information). For the GO enrichment in down regulated genes, circulatory system process was the most significant different pathway (Figure S11B, Supporting Information). We observed that there were 3420 overlapped DEGs (Figure 6D) and most of them exhibited opposite alteration trend (Figure 6E). The enrichment analysis of those overlapped DEGs with opposite regulation highlighted macrophage‐specific knockdown of Alox15 against TAD via down‐regulation of NFκB signaling pathway (Figure 6F,G).

Although BLT2 is a known receptor for 12‐HETE, the interaction between 12‐HETE and BLT2 has not been elucidated. Considering the importance of 12‐HETE and BLT2 in TAD, we performed molecular docking analysis of 12‐HETE and BLT2. This analysis showed that 12‐HETE had robust binding affinity. 12‐HETE forms intermolecular hydrogen bonds with amino acid residues of GLN, ARG, and SER and has hydrophobic interactions with PRO, VAL, ALA, LEU, and ARG in BLT2 (Figure 6H,I). Taken together, these data suggest that 12‐HETE is an essential driver of TAD development through the BLT2 receptor in mice.

**Figure 7:**
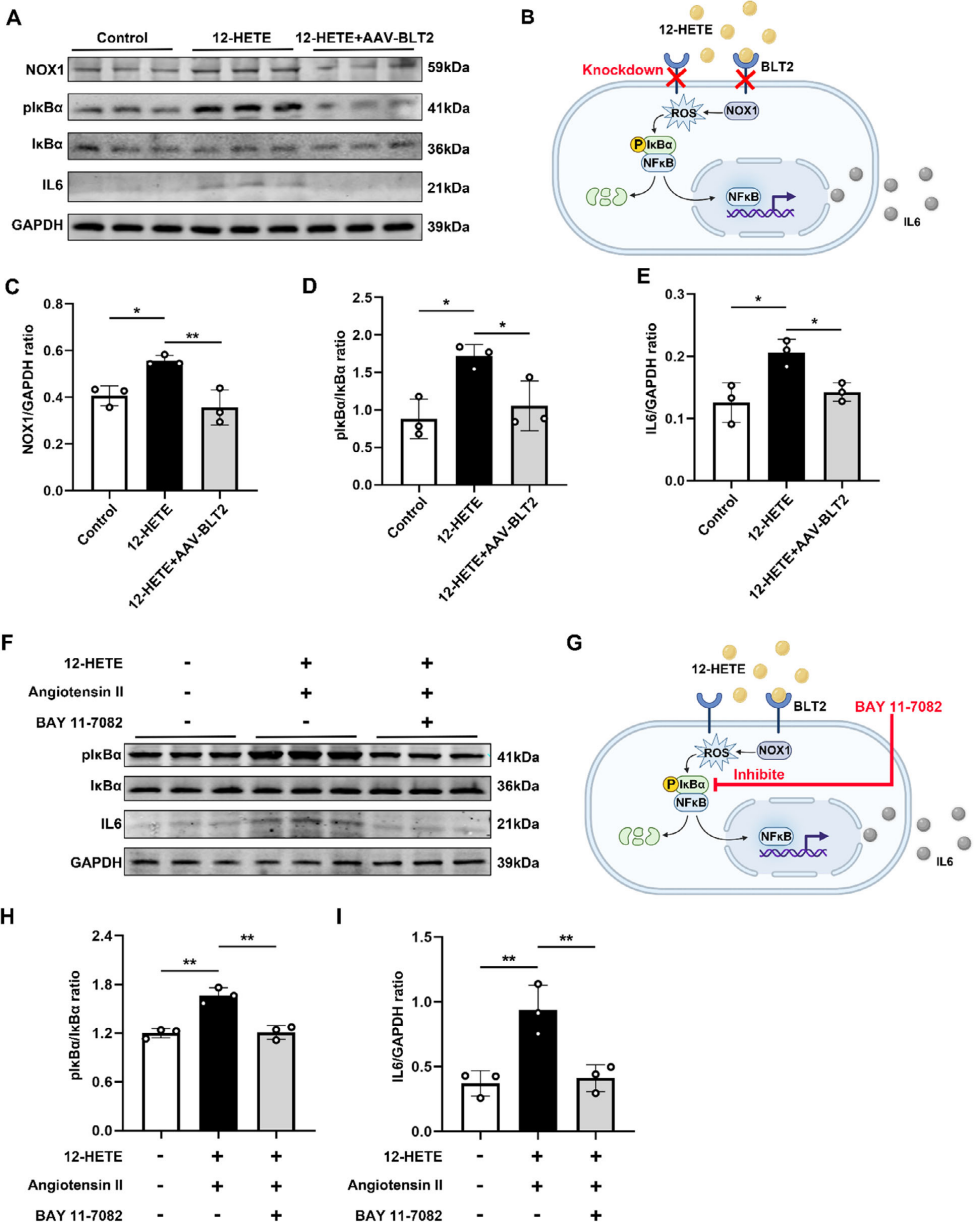
Knocking down BLT2 or inhibiting NF‐κB can reduce the production of IL‐6. A) Representative Western blot images of NOX‐1, p‐IκBα, IκBα, and IL‐6 in primary macrophages. B) Graphical abstract. Knocking down BLT2 can reduce the production of IL‐6. C–E) Western blot analysis and quantification of NOX‐1, p‐IκBα/IκBα, and IL‐6 expressed in macrophages (*n* = 3 per group). F) Representative Western blot images of p‐IκBα, IκBα, and IL‐6 in primary macrophages. G) Graphical abstract. Inhibiting NF‐κB can reduce the production of IL‐6. H,I) Western blot analysis and quantification of p‐IκBα/IκBα and IL‐6 expressed in macrophages (*n* = 3 per group). ^*^ *p* < 0.05, and ^**^ *p* < 0.01. Data were presented as the mean ± SD and analyzed by using one‐way ANOVA, Tukey's multiple comparisons test.

Because 12‐HETE and its receptor play critical roles in VSMC phenotype differentiation and inflammation onset, we explored the underlying molecular mechanism. It is unclear how the 12‐HETE–BLT2 interaction affects VSMC differentiation. We speculated that 12‐HETE binding to BLT2 activates downstream signaling. There is evidence that BLT2 regulates ROS and inflammatory factors. Primary macrophages were stimulated with 12‐HETE and/or Ang II. We observed that NOX‐1 expression was promoted upon simulation with Ang II. Upon addition of 12‐HETE, NOX‐1 expression increased (Figure 6J–L). 12‐HETE and Ang II also promote the expression of downstream p‐IκBα (Figure 6N). Finally, IL‐6 was produced in large quantities upon activation of the NOX‐1/ROS/NFκB signaling pathway (Figure 6M). We also verified the pathway by knocking down BLT2 through AAV‐BLT2, and found that the activation effect of 12HETE was also weakened (**Figure** **7**A–E). To establish a direct causal link between NF‐κB activation and IL‐6 production in our proposed pathway, we next employed a pharmacological inhibition approach. We treated primary macrophages with the specific NF‐κB inhibitor BAY 11–7082 following stimulation by 12‐HETE and Ang II. We found that inhibition of NF‐κB phosphorylation effectively suppressed the production of IL‐6 (Figure 7F–I). This result provides direct functional evidence that 12‐HETE/BLT2 signaling acts upstream of NF‐κB to induce IL‐6 release, thereby solidifying the core axis of our mechanistic model. These data suggest that 12HETE can activate the NOX1 signaling pathway through the BLT2 receptor to induce IL‐6 release.

**Figure 8:**
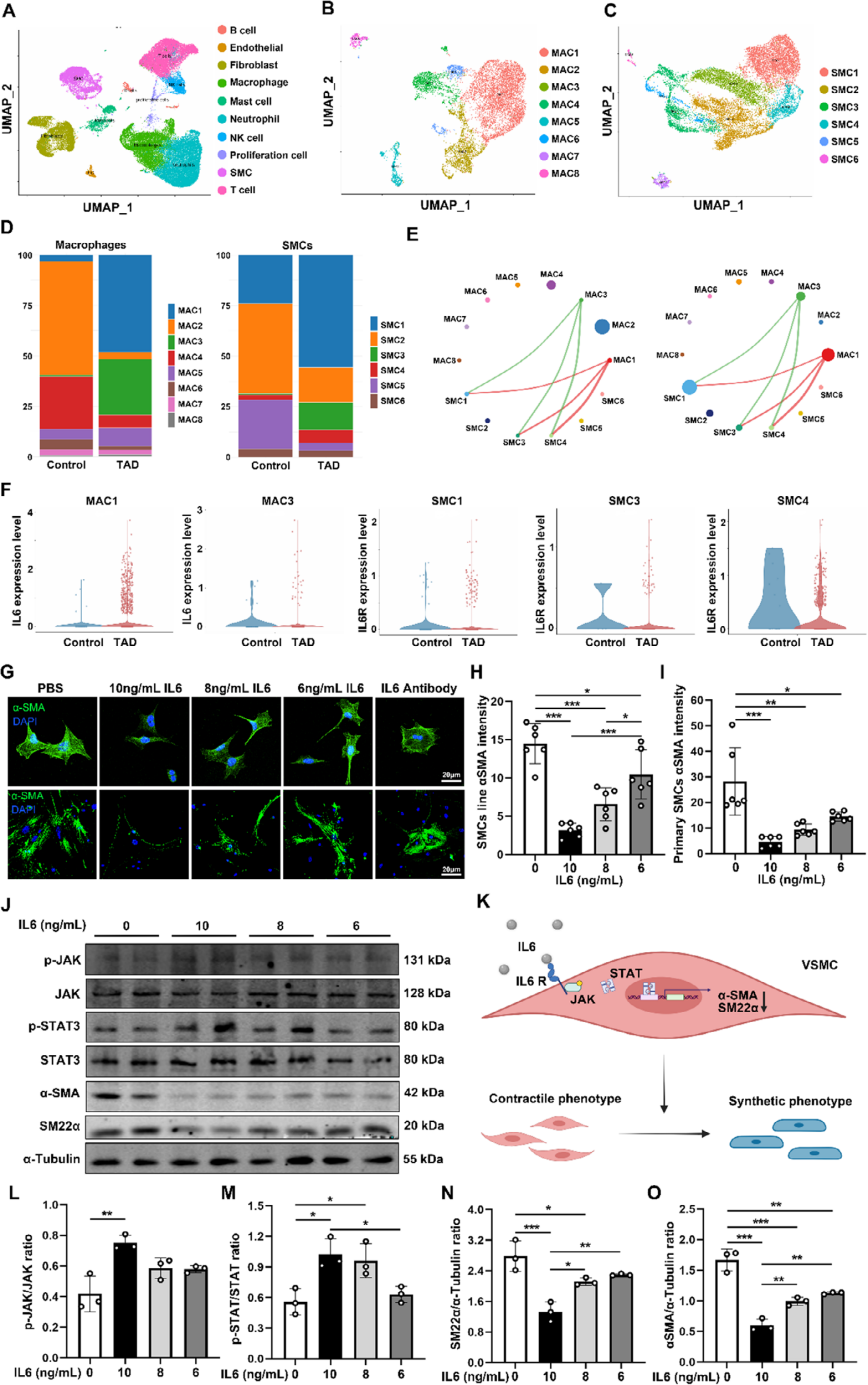
IL‐6 induced VSMC phenotypic switch through JAK/STAT signal pathway. A) UMAP plot of single‐cell clustering, showing the spatial distribution of different cell types (B cells, endothelial cells, fibroblasts, macrophages, smooth muscle cells, neutrophils, natural killer cells, and proliferating cells). B,C) UMAP plots of subpopulations of macrophages (MAC) and smooth muscle cells (SMC), with macrophages divided into MAC1 to MAC8 subpopulations and smooth muscle cells divided into SMC1 to SMC6 subpopulations. D) Proportional changes in macrophage and smooth muscle cell subpopulations across different groups, illustrating the relative differences in subpopulation proportions between the Control group and the TAD group. E) Differences in cell–cell communication between MAC1, MAC3 clusters and SMC1, SMC3, SMC4 clusters in the Control and TAD groups. Green and red arrows represent communication interactions between different cell clusters, with arrow thickness indicating communication strength. F) Differential expression of IL‐6 in macrophage and smooth muscle cell subpopulations, showing IL‐6 expression levels and statistical significance across different subpopulations in the Control and TAD groups. G) Representative confocal images of αSMA (green) in vascular smooth muscle cells and primary smooth muscle cells. (scale bar, 20 µm). H,I) Quantification of αSMA levels from confocal images (*n* = 6 per group). J) Representative Western blot images of p‐JAK, JAK, p‐STAT, STAT, αSMA, and SM22α in primary VSMCs. K) Graphical abstract. IL‐6 induced VSMC phenotypic switch though JAK/STAT. L–O) Western blot analysis and quantification of p‐JAK/JAK, p‐STAT/STAT, SM22α, and α‐SMA expressed in VSMCs (*n* = 3 per group). ^*^ *p* < 0.05, ^**^ *p* < 0.01 and ^***^ *p* < 0.001. Data were presented as the mean ± SD and analyzed by using one‐way ANOVA, Tukey's multiple comparisons test.

Furthermore, we re‐analyzed the previously published single‐cell data on aortic tissue form seven acute or subacute TAD patients and five control individuals.^[^ ^19^ ^]^ We re‐analyzed single‐cell RNA sequencing data from aortic dissection patients, dividing the dataset into a control group and a TAD group, followed by cell clustering (**Figure** **8**A; Figure S12A, Supporting Information). Subsequently, macrophages and smooth muscle cells (SMCs) underwent subgroup analysis, with macrophages categorized into MAC1‐8 and SMCs into SMC1‐6 (Figure 8B,C). Upon examining the changes in these subgroups between the control and TAD groups, we observed that in the control group, MAC2 was the most abundant subgroup, while in the TAD group, MAC1 and MAC3 showed significant increases. Similarly, in the SMC subgroups, SMC2 was predominant in the control group, whereas in the TAD group, SMC1, SMC3, and SMC4 exhibited marked increases (Figure 8D). We then analyzed the expression of IL‐6 in the increased macrophage subgroups and IL‐6R in the SMC subgroups. Both showed significant upregulation (Figure 8F). Further, we assessed cell communication strength between these subgroups in both groups and found enhanced communication between SMC1 and MAC1 in the TAD group (Figure 8E). We also examined the expression of smooth muscle contraction‐related genes, revealing a significant reduction in SMC1 in the TAD group. KEGG pathway enrichment and GSEA analysis of differential genes between SMC1 in the control and TAD groups revealed significant inhibition of smooth muscle contraction‐related pathways (Figure S12B,C, Supporting Information). To gain deeper insight into the functional state of myeloid cells within the TAD microenvironment, we performed a Gene Ontology (GO) enrichment analysis specifically on the myeloid cell cluster isolated from human TAD scRNA‐seq data. This analysis revealed a highly significant enrichment for terms related to immune activation, with “immune system process” being the most prominently overrepresented biological process (Figure S13, Supporting Information). This bioinformatic evidence directly demonstrates that myeloid cells in human TAD tissues are transcriptionally programmed to participate in immune responses, underscoring their active and potentially broad role in the pathogenesis of the disease beyond what was previously.

In order to further validate the results of the CellChat analysis, we stimulated mouse primary smooth muscle cells and VSMCs with various concentrations of IL‐6. IF staining showed that increases in IL‐6 concentrations led to reduced expression of α‐SMA in both types of smooth muscle cells (Mice VSMC cell line and primary VSMCs, Figure 8G–I). We suspected that activation of the JAK/STAT signaling pathway was associated with phenotype switching in smooth muscle cells; thus, we performed a western blot to determine the expression levels of relevant proteins. The results showed that the levels of p‐JAK/JAK and p‐STAT/STAT were substantially upregulated with 10 ng mL^−1^ of IL‐6 (Figure 8J–M). In contrast, the expression levels of αSMA and SM22α, proteins associated with phenotype switching in smooth muscle cells, were significantly downregulated (Figure 8N,O).

In addition, it was found that BLT2 receptors are also expressed in SMCs, and we extracted primary SMCs from mice and directly stimulated them with 12‐HETE, AngII, 12‐HETE+AngII. We found that smooth muscle cells significantly reduced AngII‐induced SMCs phenotypic transformation due to the addition of 12‐HETE (Figure S14, Supporting Information). In addition, other key differentiation makers (KLF4 and MYOCD) were also detected (Figure S15, Supporting Information). To functionally validate the phenotypic switch observed through marker expression, we assessed key cellular behaviors of vascular smooth muscle cells (VSMCs), including migration, proliferation, and apoptosis. Stimulation with 12‐HETE in combination with Ang II significantly inhibited VSMC proliferation and migration (Figure S16, Supporting Information). However, this treatment did not induce a significant change in the rate of apoptosis under our experimental conditions (Figure S17, Supporting Information). These functional data demonstrate that 12‐HETE primarily promotes a synthetic phenotype in VSMCs by impairing their contractile function and enhancing proliferative capacity, rather than through modulating cell survival.

### 2.8 Pharmacologic Blockade of 12/15‐LOX or Antagonism of the BLT2 Receptor Protects Mice from BAPN‐Induced TAD

**Figure 9:**
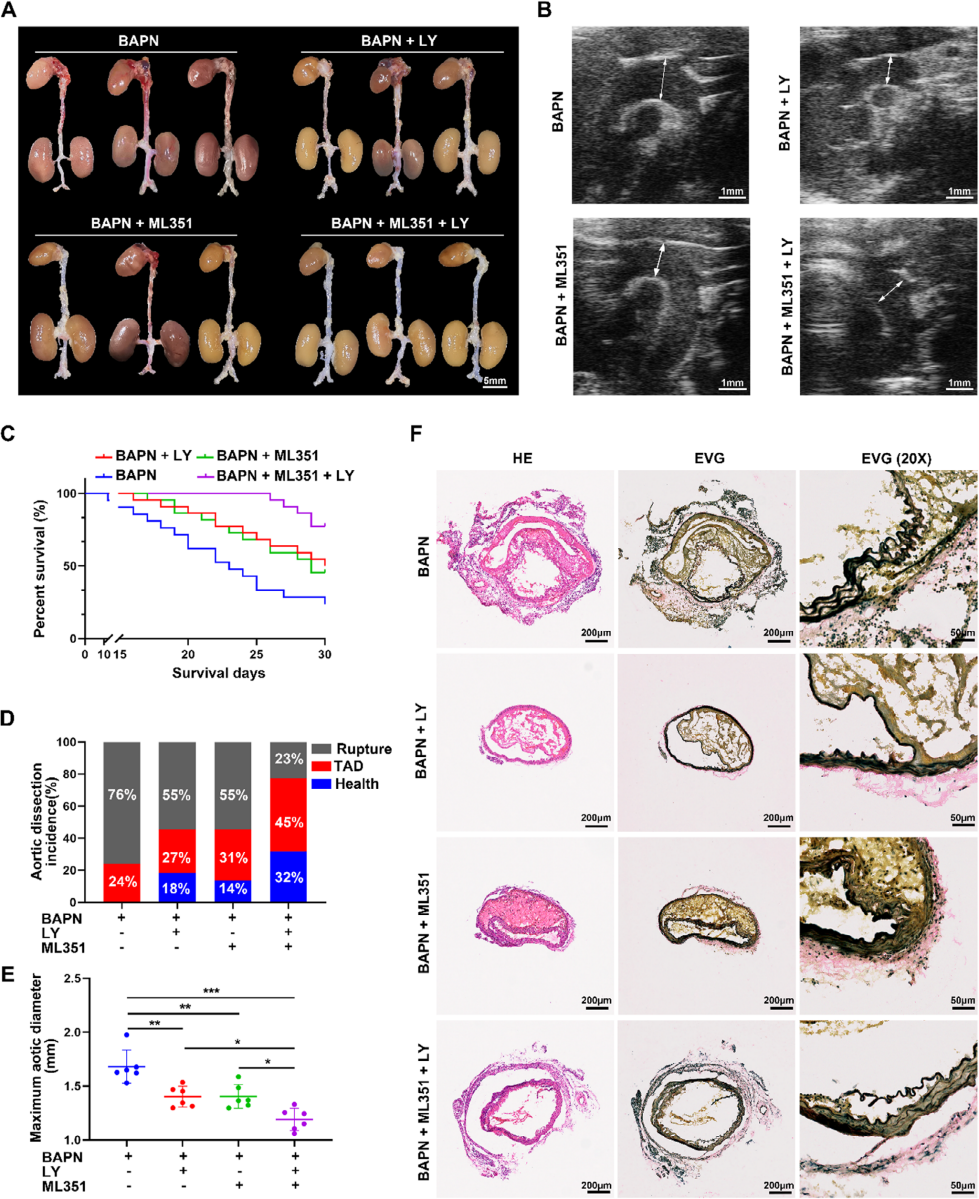
Pharmacologic blockade of Alox15 or antagonizing BLT2 receptor protects mice from BAPN‐induced TAD. A through F, C57BL/6 mice were observed with or without ML351 and LY255283 after BAPN (β‐aminopropionitrile monofumarate) treatment for 28 days. A) Representative macrographs of aorta (scale bar, 5 mm). B) Representative ultrasound images of the thoracic aorta. C) Survival rate was estimated by Kaplan–Meier method and compared by log‐rank test (*n* = 21‐22 per group). D) Thoracic aortic dissection (TAD) incidence (*n* = 21‐22 per group). E) Measurements of maximum aortic diameter (*n* = 6 per group). F) Representative macroscopic images of aorta sections stained with hematoxylin and eosin and Elastic Van Gieson (scale bars, 200; 50 µm). ^*^ *p* < 0.05, ^**^ *p* < 0.01 and ^***^ *p* < 0.001. Data were presented as the mean ± SD and analyzed by using one‐way ANOVA, Tukey's multiple comparisons test.

Through modulation of the Alox15/12‐HETE/BLT2/NF‐κB signaling pathway axis, we explored potential new targets for the clinical treatment of TAD. ML351, an inhibitor of 12/15‐LOX, can effectively reduce 12/15‐LOX expression, decreasing the plasma level of 12‐HETE. Additionally, LY255283 is a BLT2 receptor antagonist that competitively binds to elevated levels of 12‐HETE in plasma; this binding interaction hinders activation of the downstream NOX‐1/ROS/NFκB/IL‐6 signaling pathway, thereby alleviating the inflammatory response associated with TAD. We explored the therapeutic effects of ML351 and LY255283 on TAD development in a mouse model of BAPN‐induced TAD. C57BL/6J mice were intraperitoneally injected with ML351 and/or LY255283 daily after BAPN treatment throughout the 4‐week modeling period. As anticipated, the ML351 and LY255283 groups (*n* = 22) showed reduced TAD formation (**Figure** **9**A,D) and lethality (Figure 9C,D) as well as attenuated aortic dilation (Figure 9B,E), compared with the control group. When ML351 and LY255283 were used in combination, the rates of TAD incidence and rupture were greatly reduced, and the mortality rate was decreased. TAD progression was significantly inhibited. Histological detection by hematoxylin and eosin staining and Elastica van Gieson staining revealed identical pathological changes, in accordance with previous findings that 12/15‐LOX inhibition and BLT2 antagonism rescued elastin disorganization and vessel wall dissection (Figure 9F). These results revealed the central roles of 12/15‐LOX, 12‐HETE, and BLT2 in TAD progression, indicating that ML351 and LY255283 are promising drugs for the treatment of TAD.

## 3 Discussion

In this study, we observed increased levels of 12‐HETE in plasma as well as elevated 12/15‐LOX expression in diseased aortic tissue from TAD patients and TAD mice. Genetic and pharmacologic blockade of Alox15 reduced 12‐HETE released, suppressed inflammation, and VSMC phenotype switching in TAD mice. Moreover, we confirmed that 12‐HETE triggered an inflammatory cascade and facilitated VSMC phenotype switching by binding to the BLT2 receptor in macrophages and VSMCs. Thus, blockade of BLT2 prevented activation of the NOX‐1/ROS/NFκB/IL‐6 signaling pathway, downregulated VSMC differentiation markers through the JAK/STAT signaling pathway, and eventually attenuated TAD development. Our results indicate that 12‐HETE is a trigger for the inflammatory cascade, a mechanism highly relevant to aberrant VSMC phenotype switching. However, we acknowledge that systemic inflammation or metabolic alterations could represent alternative mechanisms contributing to TAD pathogenesis,^[^ ^20^ ^]^ and our study does not fully exclude these potential confounding factors. Inhibition of 12‐HETE‐related pathways (e.g., lowering plasma 12‐HETE levels or blocking its receptor) decreased TAD risk.

Arachidonic acid, an integral part of all cell membranes is released from membrane phospholipids following mechanical stimulation or stress.^[^ ^21^, ^22^ ^]^ Arachidonic acid can be metabolized into numerous metabolites via cyclooxygenase (COX), lipoxygenase (LOX), and cytochrome P450 (CYP450) with different functions and activities.^[^ ^23^ ^]^ By using targeted metabolomics analysis of eicosanoids, we identified that the active metabolites of the LOX pathway were elevated in TAD patients and mice including 12‐HETE, 8‐HETE, and 15‐HETE. Among them, 12‐HETE showed the most significant fold change. Therefore, we sought to determine the origin of 12‐HETE production in TAD patients and mice. Analysis of diseased aortic tissue revealed elevated levels of 12/15‐LOX in TAD patients and TAD mice. Accordingly, IF staining demonstrated increased 12/15‐LOX colocalization in macrophages in aorta tissue. Consistent with findings of previous single‐cell RNA‐Seq data of aortic dissection tissue harvest from BAPN‐induced TAD mice, they demonstrated that Alox15 increased in a subpopulation of macrophage as well as SMC.^[^ ^24^ ^]^ Therefore, we speculate that macrophage‐derived 12/15‐LOX is a source of the elevated plasma 12‐HETE levels in TAD, observed in response to early vascular injury though more experiments are need for further verification.

To further explore how 12‐HETE, the major metabolite of macrophage‐specific 12/15‐LOX, was involved in the development of TAD, we constructed an Alox15 knockout mouse model and determined that deletion of 12/15‐LOX decreased plasma 12‐HETE, improved survival, and reduced rupture rates. Meanwhile, 12‐HETE supplementation may aggravate aortic dissection in mice. Alox15 deficiency can promote or suppress inflammation. This is mainly due to the different downstream pathways activated by Alox15. The pro‐inflammatory pathways include the Alox15/Alox5‐mediated lipoxygenase (LOX) pathway, while the anti‐inflammatory pathways mainly include the lipoxins (LXs) pathway. In our study, we found that the deletion of Alox15 led to the infiltration of inflammatory cells and the reduction of inflammatory cytokines in aortic dissection tissues, while transcriptomic data from specific knockout mice and single‐cell data analysis showed that the activation of NF‐κB, a typical pro‐inflammatory pathway, was inhibited. Inappropriate enhanced function of Alox15 and 12‐HETE is known to trigger an inflammatory response that exacerbates organ damage.^[^ ^25^ ^]^ We found that 12‐HETE increased before inflammatory cytokines at an early stage of TAD development after 3 weeks of BAPN administration. Besides, 12‐HETE levels were positively related to inflammatory cytokines including IL‐6 in TAD, the latter rapidly activates JAK/STAT in vascular smooth muscle cells to promote an inflammatory synthetic phenotype. Therefore, 12‐HETE is paramount to the initiation and propagation of inflammation and subsequent VSMC phenotype switching in TAD development. 12‐HETE has been discovered as the endogenous ligand for BLT2.^[^ ^18^, ^26^ ^]^ Our findings confirmed the colocalization of 12‐HETE with BLT2 in macrophages, ECs, and VSMCs. The binding of 12‐HETE to BLT2 results in the activation of BLT2, thereby stimulating the activation of the NOX‐1/ROS/NFκB/IL‐6 signaling pathway in macrophages, ECs, and VSMCs. Furthermore, we acknowledge that BLT2 is widely expressed in the TAD aortic wall. Although our study focused on its role in macrophages, endothelial cells, and smooth muscle cells, other immune cells such as neutrophils, which significantly infiltrate the aortic tissue in TAD, also express BLT2. The potential contribution of these cells to the 12‐HETE/BLT2 signaling cascade represents an important area for future investigation. The BLT2 inhibitor LY255283 reversed the protective effect of Alox15 deletion on TAD progression. BLT2 is a known member of the LTB4 family.^[^ ^27^, ^28^ ^]^ However, without the available structure of BLT2, their specific binding sites and mechanism of action remain unclear. We use AlphaFold model to predict the protein structure of BLT2 and examinate the binding affinity and mode of binding between 12‐HETE and BLT2 using molecular docking analysis. Collectively, we identified putative binding sites for 12‐HETE and BLT2 and provides evidence that 12‐HETE activated the downstream NOX‐1/ROS/NFκB/IL‐6 signaling pathway, leading to inflammatory factor production and promoting VSMC phenotype switching via BLT2 activation.

While our findings establish a central role for the 12‐HETE/BLT2 axis, we recognize that other pathways and cell types may contribute to TAD pathogenesis. For instance, the COX and CYP450 pathways of arachidonic acid metabolism, which produce various bioactive lipids, were not thoroughly investigated in this study and may represent parallel or compensatory mechanisms. Additionally, while our data highlight macrophages and VSMCs as key players, the potential involvement of other cell types such as fibroblasts and T‐cells warrants further exploration. Our study identified that 12‐HETE initiates the inflammatory cascade and triggers aberrant phenotype switching in VSMCs via BLT2 receptor during TAD development and shows potential as a biomarker of TAD. However, further validation of its clinical utility in TAD risk prediction is still required in the following clinical trials in cohorts. Besides, to our knowledge, at this point, it is still unclear which BLT2 domain binds 12‐HETE. Although molecular docking experiments were performed to predict the binding site of 12‐HETE to a predicted BLT2 protein structure by Alphafold, our result needs further confirmation in cryo‐electron microscopy ultrastructural studies. The combination of the 12/15‐LOX inhibitor and BLT2 blocker greatly reduces aortic injury in TAD mice. But the chronic safety and systematic influence of both inhibitors should be investigated before considering the clinical success of TAD prevention by pharmaceutically targeting this target.

Regarding the specific methodology for macrophage‐specific deletion of Alox15, it should be noted that the AAV9‐CD68 system was used in this study as an approach to generate supportive evidence. While this strategy provided valuable insights, we acknowledge that the use of Alox15‐floxed mice crossed with Lyz2‐Cre mice represents the genetically most precise method for achieving macrophage‐specific knockout. Thus, employing such conditional knockout models to further validate the findings remains an important objective for future research.

In our study, we found that neutrophils and macrophages infiltrated significantly in WT mice with aortic dissection, while A lox15 KO significantly ameliorated this phenomenon. Through our omics sequencing of the aorta of macrophage Alox15‐specific knockout mice, it was found that in aortic dissection, the deletion of Alox15 mainly alleviated the NF‐κB signaling pathway. In human aortic dissection single‐cell data, we found that macrophage NF‐κB pathway with high Alox15 expression in aortic dissection tissue was also activated. Therefore, we hypothesized that the deletion of Alox15 would lead to the downregulation of the NF‐κB signaling pathway, which would lead to a decrease in the release of pro‐inflammatory factors by macrophages, thereby further reducing the amplification effect of macrophage‐induced inflammatory cascade in aortic dissection. This is further validated by our bone marrow and blood flow cytometry.

The present study demonstrated the effects of 12/15‐LOX and its metabolite 12‐HETE on TAD development. During TAD progression, 12‐HETE triggers the initiation of an inflammatory cascade and substantial VSMC differentiation through binding to BLT2 on macrophages. Targeted inhibition of 12/15‐LOX and blockade of BLT2 may be useful in the development of novel therapeutic strategies. Nevertheless, the complex interplay between different inflammatory pathways and cell types in TAD suggests that a multifaceted therapeutic approach might be necessary for optimal clinical outcomes.

## 4 Experimental Section

### Human Plasma Samples

Human plasma samples used in this study were collected from TAD patients in Beijing Anzhen Hospital. Immediately after collection using EDTA‐coated tubes, samples were placed on ice and processed within 30 min through two‐step centrifugation (3000 rpm for 15 min at 4°) to obtain platelet‐poor plasma. Processed aliquots were flash‐frozen in liquid nitrogen and stored at ‐80°until analysis. Both control and TAD groups were processed simultaneously using identical protocols to minimize batch effects. The clinical characteristics of the study participants are listed in Table S1 (Supporting Information). All samples were only used for research purposes; written informed consent was obtained from donors or their families, in accordance with Chinese regulations concerning the collection of informed consent. All protocols and procedures involving human samples were approved by the Beijing Anzhen Hospital Review Board and conducted in accordance with principles established in the Declaration of Helsinki. Patients were recruited as part of the DPANDA registry study for aortic aneurysm and/or dissection (ClinicalTrials.gov: NCT03233087).

### Targeted Arachidonic Acid Pathway Metabolites Assay

Plasma samples (200 µL) were subjected to lipid extraction using a methanol‐based buffer containing 0.1% (w/v) butylated hydroxytoluene (BHT) and butylated hydroxyanisole (BHA), along with formic acid and a comprehensive internal standard cocktail (Cayman Chemicals, USA). The internal standards included deuterated analogs of key eicosanoids such as PGD2‐d4, PGE2‐d4, 5(S)‐HETE‐d8, 12(S)‐HETE‐d8, 15(S)‐HETE‐d8, leukotriene B4‐d4, and others, covering prostaglandins, hydroxyeicosatetraenoic acids (HETEs), epoxyeicosatrienoic acids (EETs), dihydroxyoctadecenoic acids (DiHOMEs), and related metabolites. After vortexing, samples were incubated at 4 °C for 12 h with continuous shaking at 1500 rpm. Subsequently, the mixtures were centrifuged at 12 000 rpm for 10 min at 4 °C, and the supernatant was collected. The extraction procedure was repeated once to improve metabolite recovery. The combined supernatants were then purified and enriched by solid‐phase extraction (SPE) using Oasis Prime HLB columns (30 mg, Waters, USA). To prevent complete drying, SPE eluents were collected in tubes pre‐loaded with 20 µL of an ethanol‐glycerol (1:1, v/v) mixture and dried under a gentle stream of nitrogen. The dried extracts were immediately reconstituted in 50 µL of water: acetonitrile: formic acid (63:37:0.02, v/v/v) prior to LC‐MS analysis. Eicosanoid profiling was performed on an ExionLC‐AD system coupled to a Sciex QTRAP 6500 Plus mass spectrometer (Sciex, USA). Separation was achieved on a Phenomenex Kinetex C18 column (100 × 2.1 mm, 1.7 µm) with mobile phase A consisting of water: acetonitrile: formic acid (63:37:0.02, v/v/v) and mobile phase B comprising acetonitrile: isopropanol (1:1, v/v).

### Animals

All procedures with animals were approved by the Committee on the Ethics of Animal Experiments of Capital Medical University and performed in accordance with the guidelines from Directive 2010/63/EU of the European Parliament. After the study, all animals were anesthetized by isoflurane inhalation (1.5–2%) and then euthanized by cervical dislocation. Further details regarding the animals used can be found in (Figure S18A,B, Supporting Information). In global 12/15‐LOX knockout mice (Alox15^−/v^), plasma 12‐HETE (Figure S18C, Supporting Information), 15‐HETE (Figure S18D, Supporting Information), and LOX (Figure S18E, Supporting Information) levels were significantly downregulated.

All animal experiments were conducted in compliance with the National Institutes of Health Guidelines for the Protection and Use of Laboratory Animals and were approved by the Ethical Review Committee of Beijing Anzhen Hospital. Both male and female mice were used in this study (with sex‐specific analyses reported in Results). WT and Alox15^−/−^ mice on the C57BL/6J background were used, including global 12/15‐LOX knockout mice purchased from the Jackson Laboratory and generated in the Animal Laboratory of Beijing Anzhen Hospital, and WT mice purchased from Beijing Huafukang Biotechnology Co., Ltd. All mice were housed under temperature‐controlled conditions (22 ± 2 °C) with a 12 h light/dark cycle, provided with standard rodent maintenance diet (AIN‐93G, Beijing HFK Bioscience Co.) and filtered water ad libitum.For experimental procedures, mice were randomly assigned to treatment groups using computer‐generated randomization. Investigators were blinded to group allocation during data collection and analysis. 4‐week‐old WT and Alox15^−/−^ mice received BAPN (1 g kg^−1^ day^−1^, Sigma–Aldrich) in drinking water for 4 weeks to induce aortic dissection. Experimental groups received daily intraperitoneal injections of either 12‐HETE (27 µg kg^−1^ day^−1^), ML351 (10 mg kg^−1^ day^−1^), or LY255283 (3 mg kg^−1^ day^−1^) throughout the 4‐week period. Vehicle control groups received equivalent volumes of PBS with 0.1% DMSO. All compounds were prepared fresh daily and sterile filtered (0.22 µm). Animal welfare was monitored twice daily, with particular attention to potential drug side effects including weight loss (> 15% body weight threshold), reduced activity, or neurological symptoms. No severe adverse effects were observed at the administered doses. At the endpoint, mice were anesthetized with 1% pentobarbital sodium (50 mg kg^−1^ i.p.) prior to euthanasia and tissue collection.

### IF Staining

Formaldehyde‐fixed cells or frozen tissue sections were permeabilized with 0.3% Triton X‐100 and blocked with 5% bovine plasma albumin, then incubated overnight at 4 °C with the specific primary antibodies listed in Table S3 (Supporting Information). After several washes with phosphate‐buffered saline (PBS), cells were incubated for 1–2 h at room‐temperature with the fluorescent secondary antibodies listed in Table S4 (Supporting Information). 4′,6‐diamidino‐2‐phenylindole (DAPI; Sigma–Aldrich) was added and incubated with the cells for 5 min before observation. IF was visualized using a confocal microscope (LSM 710, Zeiss, Oberkochen, Germany). For each image, the number of positive cells and total cell nuclei were quantified, and the percentage of positive cells was calculated.

### Hematoxylin and Eosin Staining and Elastica Van Gieson

Aortic sections were stained with hematoxylin and eosin (Sigma–Aldrich) and Elastica Van Gieson reagents (Sigma–Aldrich), in accordance with the manufacturer's instructions. For hematoxylin and eosin staining, tissue sections were completely covered with Mayer's hematoxylin (Lillie's modification) and incubated for 5 min. Next, slides were rinsed twice with distilled water to remove excess stain. Subsequently, tissue sections were completely covered with Bluing Reagent and incubated for 10–15 s. Slides were then rinsed twice with distilled water. Slides were immersed in absolute alcohol and blotted dry. Tissue sections were completely covered with eosin Y solution (modified alcoholic), incubated for 2–3 min, and rinsed with absolute alcohol. Slides were dehydrated three times in absolute alcohol. Finally, slides were cleared and mounted with synthetic resin.

For Elastica Van Gieson, slides were immersed in working Elastic Stain Solution for 15 min, then rinsed with running tap water until excess stain had been removed. Next, slides were immersed 15–20 times in Differentiating Solution and rinsed with tap water. The slides were checked under a microscope to ensure proper differentiation. Subsequently, slides were rinsed with running tap water, placed in sodium thiosulfate solution for 1 min, then rinsed in running tap water again. Slides were stained with Elastica Van Gieson Esolution for 2–5 min, then rinsed twice with 95% alcohol and dehydrated in absolute alcohol. Finally, slides were cleared and mounted with synthetic resin. Images were acquired with an Olympus DP73 microscope digital camera (Olympus, Tokyo, Japan).

### Quantitative Reverse Transcription (RT)‐PCR

Total RNA was extracted from aortic tissue using Trizol reagent (Invitrogen), in accordance with the manufacturer's instructions. RNA samples (1 µg) were subsequently reverse‐transcribed into cDNA with a Reverse Transcription Reagent Kit (Bio‐Rad), and the resulting cDNA was amplified by semi‐quantitative RT‐PCR using SYBR Green Mix (Applied Biosystems, Waltham, MA, USA). mRNA levels were determined from cycle threshold (Ct) values and normalized against the Ct of 18S rRNA or β‐actin. All primers used for RT‐PCR are listed in Table S3 (Supporting Information).

### Flow Cytometry Analysis of Blood, Bone Marrow, and Spleen

Leukocytes, including neutrophils, monocytes, and lymphocytes, were identified in whole blood, bone marrow (BM), and spleen as previously described, with minor modifications. Briefly, fresh mouse blood was collected from the tail vein using heparin‐coated capillary tubes and immediately transferred to ice‐cold microcentrifuge tubes containing ethylenediaminetetraacetic acid (EDTA; 5 mm). A small aliquot was reserved for plasma separation and complete blood count analysis. The remaining blood was subjected to red blood cell (RBC) lysis using BD Pharm Lyse buffer (BD Biosciences, San Jose, CA, USA), followed by washing and resuspension in PBS or FACS buffer (Hanks' balanced salt solution supplemented with 0.1% w/v bovine plasma albumin and 5 mm EDTA) for antibody staining.

For BM isolation, femurs and tibias were dissected, cleaned, and cut open to expose the marrow cavity. BM was flushed with ice‐cold PBS using a 23‐gauge needle and a 10 mL syringe, passed through a 40 µm nylon mesh strainer, and collected in a 50 mL tube. The cell suspension was centrifuged at 300 × g for 10 min at 4 °C. After supernatant removal, RBCs were lysed, and the remaining cells were washed twice with PBS.

Spleens were weighed, mechanically dissociated using the plunger end of a 1 mL syringe, and filtered through a 40 µm mesh to generate single‐cell suspensions. After centrifugation (300 × g, 10 min, 4 °C), cells were treated with RBC lysis buffer, washed, and counted.

To ensure accurate population identification, single‐color compensation controls were prepared using UltraComp eBeads (Thermo Fisher Scientific) or unstained cells, and fluorescence minus one (FMO) controls were used to guide gating strategies for multicolor panels. Cells were resuspended in 100 µL FACS buffer containing a cocktail of fluorochrome‐conjugated antibodies targeting leukocyte surface markers and incubated for 30 min at 4 °C in the dark. After staining, cells were washed and resuspended in FACS buffer for acquisition.

Samples were analyzed on an LSRII flow cytometer (BD Biosciences) using FACSDiva software, or an ImageStreamX Mark II imaging flow cytometer (Luminex) with IDEAS 6.2 software. Data analysis was performed using FlowJo software (BD Life Sciences). Macrophages were defined as CD45⁺CD11b⁺F4/80⁺ and further subdivided into CD206⁺ or CD206^−^ subsets; neutrophils were identified as CD45⁺CD11b⁺Ly6G^hi^.

### Enzyme‐Linked Immunosorbent Assays

Cytokine concentrations in mouse plasma were quantified using commercial uncoated ELISA kits. Briefly, blood samples were centrifuged at 3000 × g for 10 min to separate plasma, which was immediately aliquoted and stored at ‐80 °C until analysis. The levels of interleukin‐1β (IL‐1β), tumor necrosis factor‐alpha (TNF‐α), interleukin‐6 (IL‐6), and monocyte chemoattractant protein‐1 (CCL2) were measured using specific mouse ELISA kits (Shandong Sparkjade Biotechnology Co., Ltd., China) according to the manufacturer's protocols.

The assay sensitivities, defined as the minimum detectable concentration, were as follows: 1.5 pg mL^−1^ for IL‐1β, 3.0 pg mL^−1^ for TNF‐α, 2.0 pg mL^−1^ for IL‐6, and 5.0 pg mL^−1^ for CCL‐2. All samples were assayed in duplicate, and the average of the two measurements was used for statistical analysis. The intra‐assay coefficients of variation (CV) for each analyte were confirmed to be below 8%, ensuring reproducibility and precision of the measurements. Absorbance was read at 450 nm using a microplate reader, and cytokine concentrations were calculated based on the standard curves generated for each assay.

### RNA‐seq

For transcriptomic profiling, aortic tissues were harvested from three experimental groups: 1) Control (untreated wild‐type mice), 2) AAV‐empty (mice subjected to BAPN‐induced aortic dissection and administered empty AAV vector), and 3) AAV‐Alox15 (BAPN‐induced mice treated with AAV‐mediated Alox15 overexpression). Tissues were immediately snap‐frozen in liquid nitrogen and stored at ‐80 °C until processing. Total RNA was extracted using the RNeasy Mini Kit (Qiagen) with on‐column DNase I digestion to eliminate genomic DNA contamination. RNA integrity was verified by Agilent Bioanalyzer (RIN > 7.0), and quantification was performed using Qubit fluorometric analysis.

For library preparation, 1 µg of high‐quality total RNA per sample underwent poly(A)+ mRNA selection using NEBNext Poly(A) mRNA Magnetic Isolation Module. Strand‐specific cDNA libraries were constructed with the NEBNext Ultra II Directional RNA Library Prep Kit, incorporating unique dual‐index adapters for sample multiplexing. Library quality was assessed by Bioanalyzer, and quantification was performed via qPCR (Kapa Biosystems). Pooled libraries were sequenced on an Illumina NovaSeq 6000 platform (150 bp paired‐end reads) to achieve a minimum depth of 40 million reads per sample.

Bioinformatic analysis began with raw read quality control using FastQC and multiqc. Adapter trimming and quality filtering were performed with Trim Galore! (cutoff Q30). Cleaned reads were aligned to the mm10 mouse reference genome using the STAR aligner with two‐pass mode for improved splice junction detection. Gene‐level quantification was obtained using featureCounts (Subread package) with GENCODE annotation. Differential expression analysis between groups was conducted in R using DESeq2 with adjusted *p*‐value < 0.05 and |log2FC| > 1 as significance thresholds. Functional enrichment analysis of differentially expressed genes was performed using clusterProfiler for GO terms and KEGG pathways, with GSEA employed for pathway‐level analysis. Cell‐type deconvolution was performed using CIBERSORTx to assess potential changes in aortic cellular composition. Quality metrics including PCA, sample clustering, and correlation heatmaps were generated to ensure data reproducibility.

### Single Cell RNAseq

The publicly available dataset (GSE222318) is reprocessed using the Seurat package (v4.1.0) in R software (v4.1.2). The data processing pipeline included: first, normalization and batch correction were performed following the cell annotation method consistent with the original publication [Citation]. Next, the RunUMAP function is used to generate a Uniform Manifold Approximation and Projection (UMAP) for dimensionality reduction, providing a foundation for downstream cell clustering analysis. Cell clustering was performed using the FindNeighbors and FindClusters functions to identify distinct cell types. Based on these analyses, the pattern recognition module in the CellChat package is run for both TAD and Control group samples, utilizing its built‐in ligand‐receptor interaction database (CellChatDB) to perform intercellular communication analysis.

### Western Blot Analysis

Western blotting was performed to investigate the expression of key signaling proteins. Total proteins were extracted from tissues or cells using RIPA lysis buffer supplemented with protease and phosphatase inhibitors. Protein concentrations were determined using the BCA assay. Equal amounts of protein (30–50 µg per lane) were separated by 10% or 12% SDS‐polyacrylamide gel electrophoresis and subsequently transferred onto PVDF membranes. The membranes were blocked with 5% non‐fat milk in TBST for 1 h at room‐temperature and then incubated overnight at 4 °C with the following primary antibodies: NOX1 (Proteintech, 17772‐1‐AP, 1:1000), p‐IκBα (Santa Cruz, sc‐8404, 1:1000), IκBα (Santa Cruz, sc‐1643, 1:1000), IL‐6 (Santa Cruz, sc‐35596, 1:1000), p‐JAK (Abclonal, AP0531, 1:1000), JAK (Santa Cruz, sc‐390539, 1:1000), p‐STAT (CST, 9145S, 1:1000), STAT (CST, 9139S, 1:1000), Collagen I (Abclonal, A16891, 1:1000), α‐SMA (CST, 48938S, 1:1000), SM22α (Santa Cruz, sc‐53932, 1:1000), α‐Tubulin (CST, 2144S, 1:1000), and GAPDH (CST, 2118S, 1:1000).

After washing, the membranes were incubated with appropriate horseradish peroxidase‐conjugated secondary antibodies for 1 h at room‐temperature. Protein bands were visualized using an enhanced chemiluminescence detection system. Band intensities were quantified by densitometric analysis using ImageJ software (National Institutes of Health, USA), and the expression levels of target proteins were normalized to those of α‐Tubulin or GAPDH as internal controls.

### Molecular Docking

The 2D molecular structure of 12(S)‐HETE was downloaded from PubChem (https://pubchem.ncbi.nlm.nih.gov, PubChem CID: 5283155), and minimized in Discovery Studio ver. 4.5. The 3D structure of the protein LT4R2 was retrieved from the Alpha Fold Protein Structure Database (https://alphafold.ebi.ac.uk/; Human code Q9NPC1). AutoDockTools ver. 1.5.6 was used to convert 12‐HETE and BLT2 protein molecules into “pdbqt” format, and finally vina was used for molecular docking.

### Statistical Analysis

Data are presented as the mean ± SD. Statistical analysis was performed using GraphPad Prism software (version 8.02). An unpaired two‐tailed Student's *t*‐test was used for comparisons between two groups. For comparisons among more than two groups, one‐way analysis of variance (ANOVA) with Tukey's post hoc test was applied. The non‐parametric Kruskal–Wallis test followed by Dunn's multiple comparisons test was used for analyzing elastin break grades. Survival curves were generated by the Kaplan‐Meier method and compared with the log‐rank test. Correlation analysis was performed using linear regression and Spearman's method. Statistical significance was defined as ^*^ *p* < 0.05, ^**^ *p* < 0.01, and ^***^ *p* < 0.001.

## Conflict of Interest

The authors declare no conflict of interest.

## Supporting information



Supporting Information

## Data Availability

The data that support the findings of this study are available from the corresponding author upon reasonable request.
