# Supplementary material for: 12‐HETE is an Endogenous Modulator of BLT2 Triggering Vascular Degeneration, Dissection, and Rupture
Source: Adv Sci (Weinh). 2025 Nov 5;13(5):e15897. doi: 10.1002/advs.202515897 (PMC12849872; doi:10.1002/advs.202515897)
Supplement: Supplementary file 1 — Supporting Information [file ADVS-13-e15897-s001.docx]

# SUPPLEMETARY FIGURES


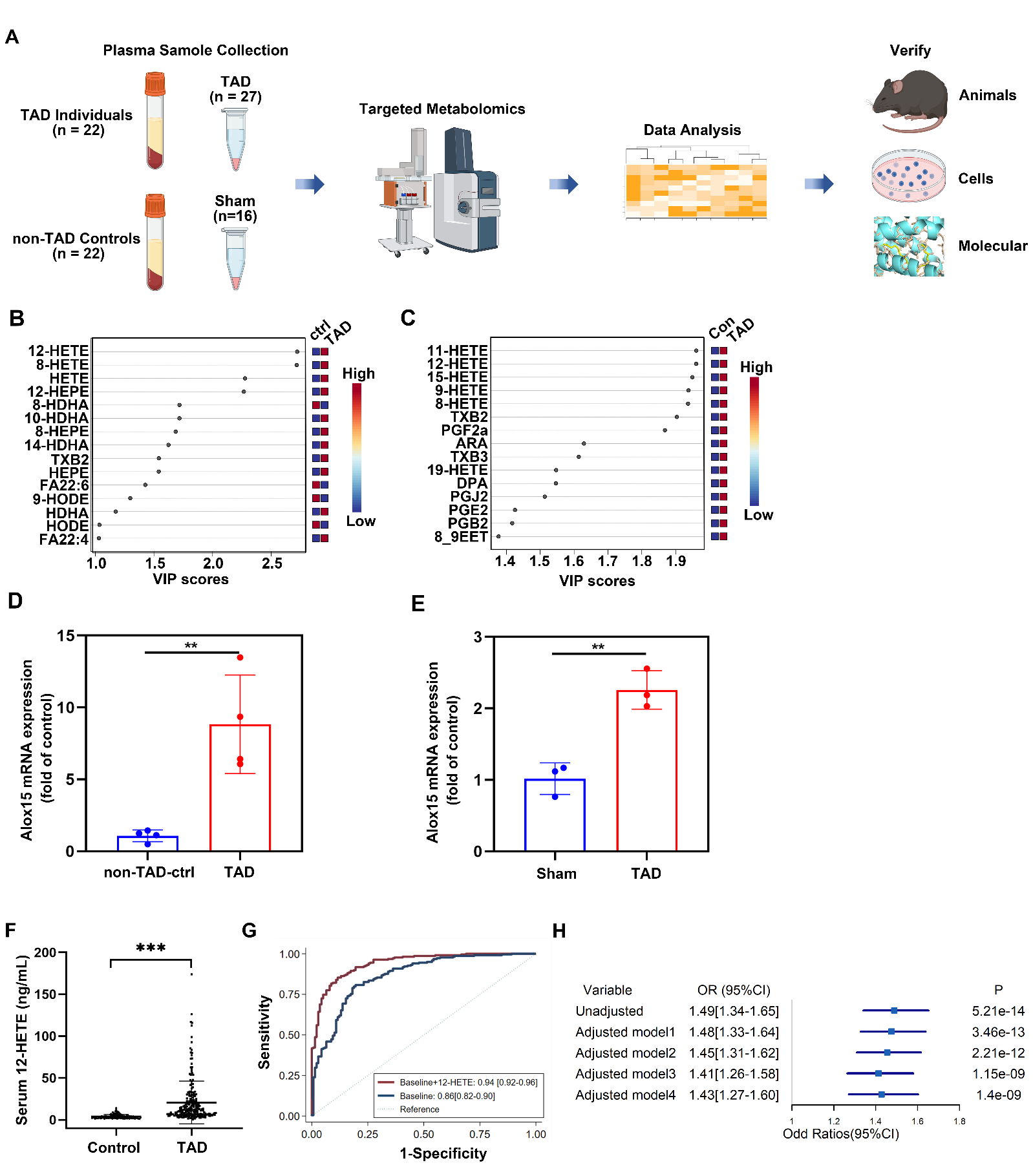


Figure S1**. A**, Schematic showing the metabolomics-based experimental design using plasma samples collected from individuals with TAD and BAPN-induced TAD mouse. **B** and **C**, Features (variables) of top 15 most significant metabolites based on VIP scores from PLS-DA. The x-axis shows correlation scores and the y-axis shows the metabolites. **D** and **E**, The mRNA expression level of Alox15 in the aorta of individuals with TAD and BAPN-induced TAD mouse. **F**, Plasma 12HETE concentration of TAD individuals and non-TAD controls in Pilot study. **G**, Receiver Operating Characteristic (ROC) curve showing the performance of different individuals (TAD, non-TAD) in terms of true positive rate and false positive rate. **H**, Forest plot illustrates that plasma 12-HETE indepently distinguish AAD from controls. **p* < 0.05, ***p* < 0.01 and ****p* < 0.001. Data were presented as the mean±SD and analyzed by using an unpaired two-tailed Student’s t test.


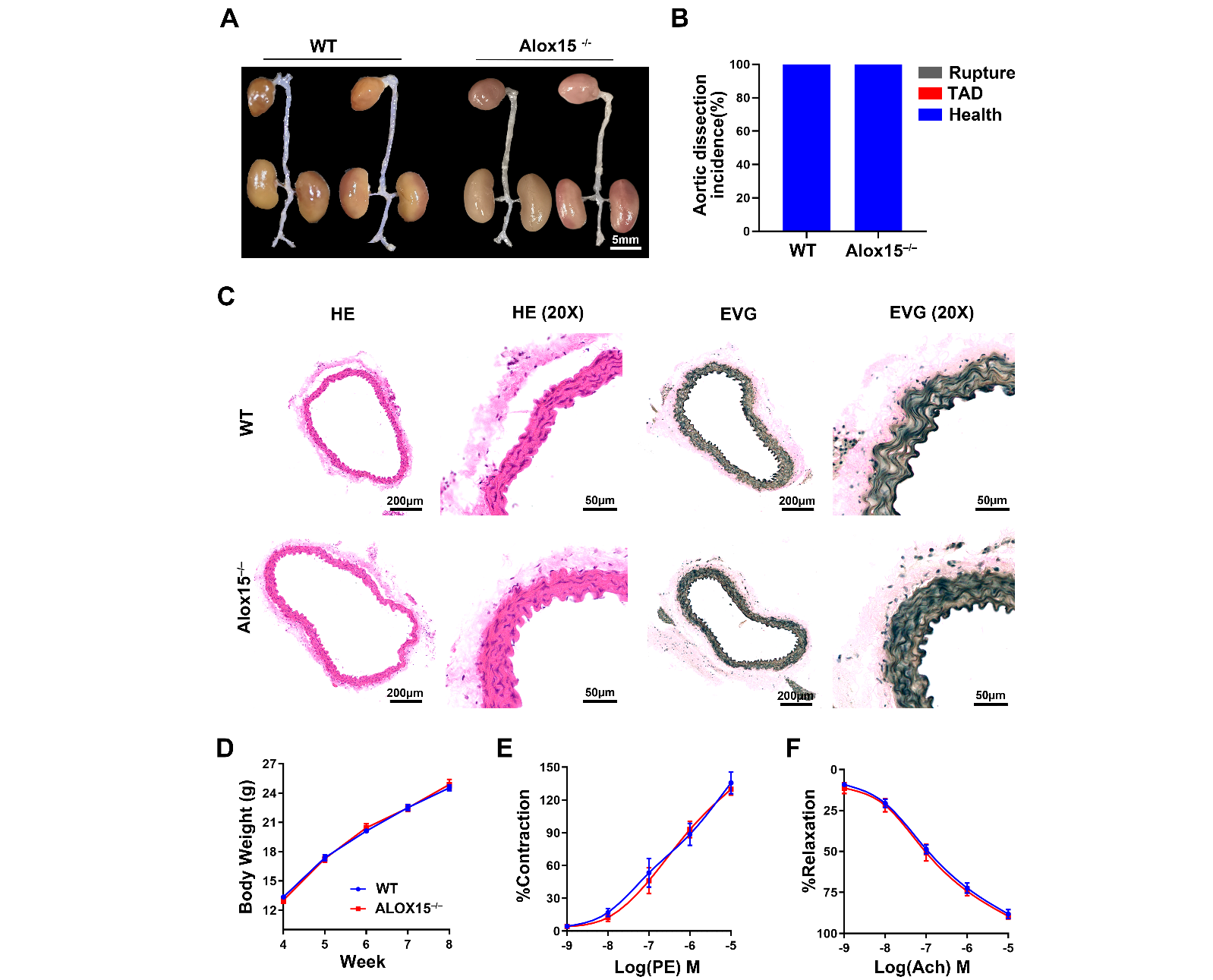


Figure S2**. A**, Presentative macrographs of aorta from WT and Alox15^–/–^ mice, scale bars = 5mm. **B**, TAD incidence of WT and Alox15^–/–^ mice (n=6 per group). **C**, Representative macroscopic images of aorta sections stained with hematoxylin and eosin (HE) and Elastic Van Gieson (EVG) from WT and Alox15^–/–^ mice. Scale bars: 200μm and 50μm. **D**, Body weight (g) changes of WT and Alox15^–/–^ mice for 28 days (n=6 per group). **E** and **F**, Acetylcholine (Ach)-induced endothelium-dependent vasodilation and phenylephrine (PE)-induced vascular contraction of aortic rings from WT and Alox15^–/–^ mice (n=6 per group).


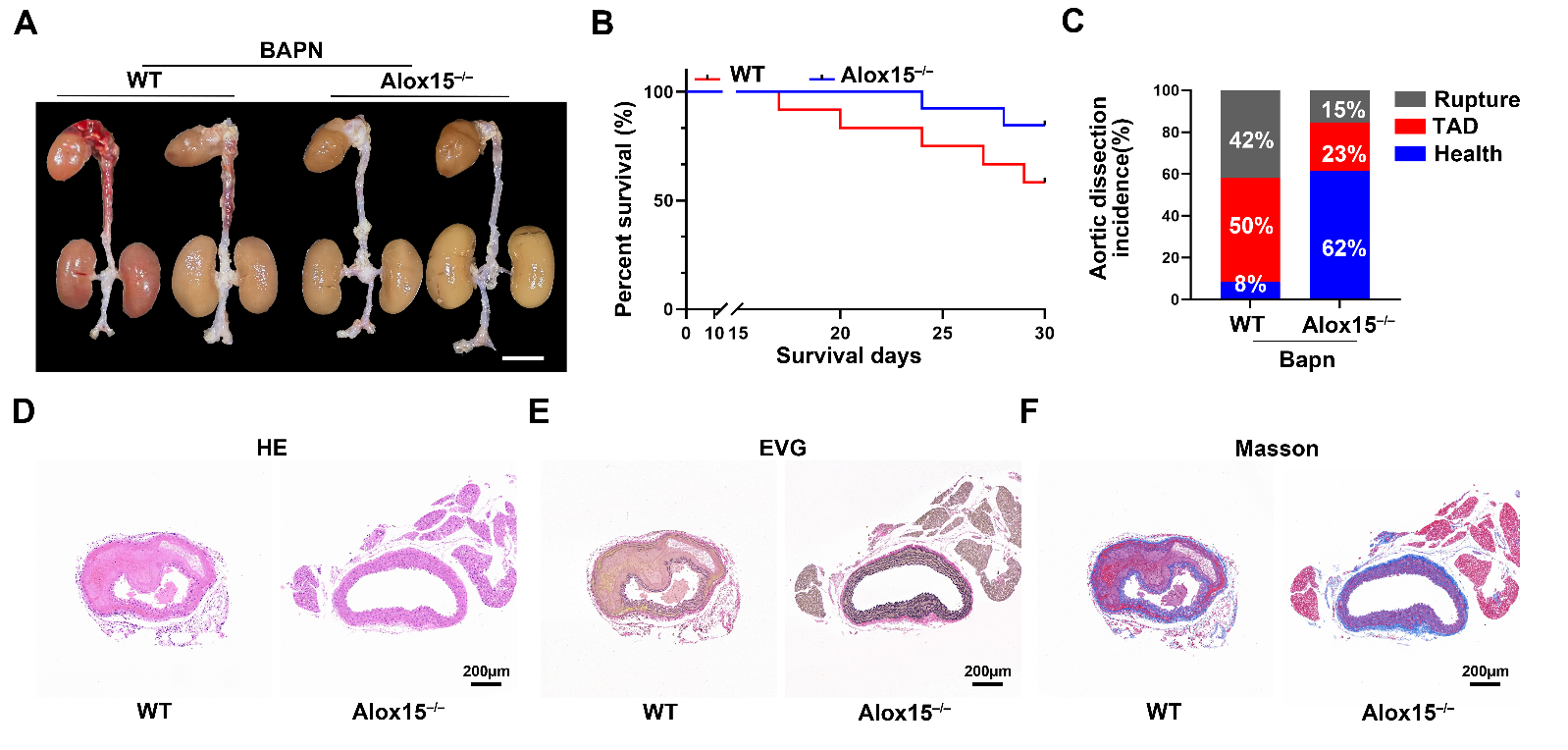


Figure S3**.** Female C57BL/6J mice were observed with or without Alox15^–/–^ after BAPN (β-aminopropionitrile monofumarate) treatment for 28 days. **A,** Representative macrographs of aorta (scale bar, 5 mm). **B,** Survival rate was estimated by Kaplan-Meier method and compared by log-rank test (n=12 and 13 per group). **C,** Thoracic aortic dissection (TAD) incidence (n=12 and 13 per group). **D-F,** Representative macroscopic images of aorta sections stained with hematoxylin and eosin (HE), Elastic Van Gieson (EVG) and Masson (scale bars, 200 μm).


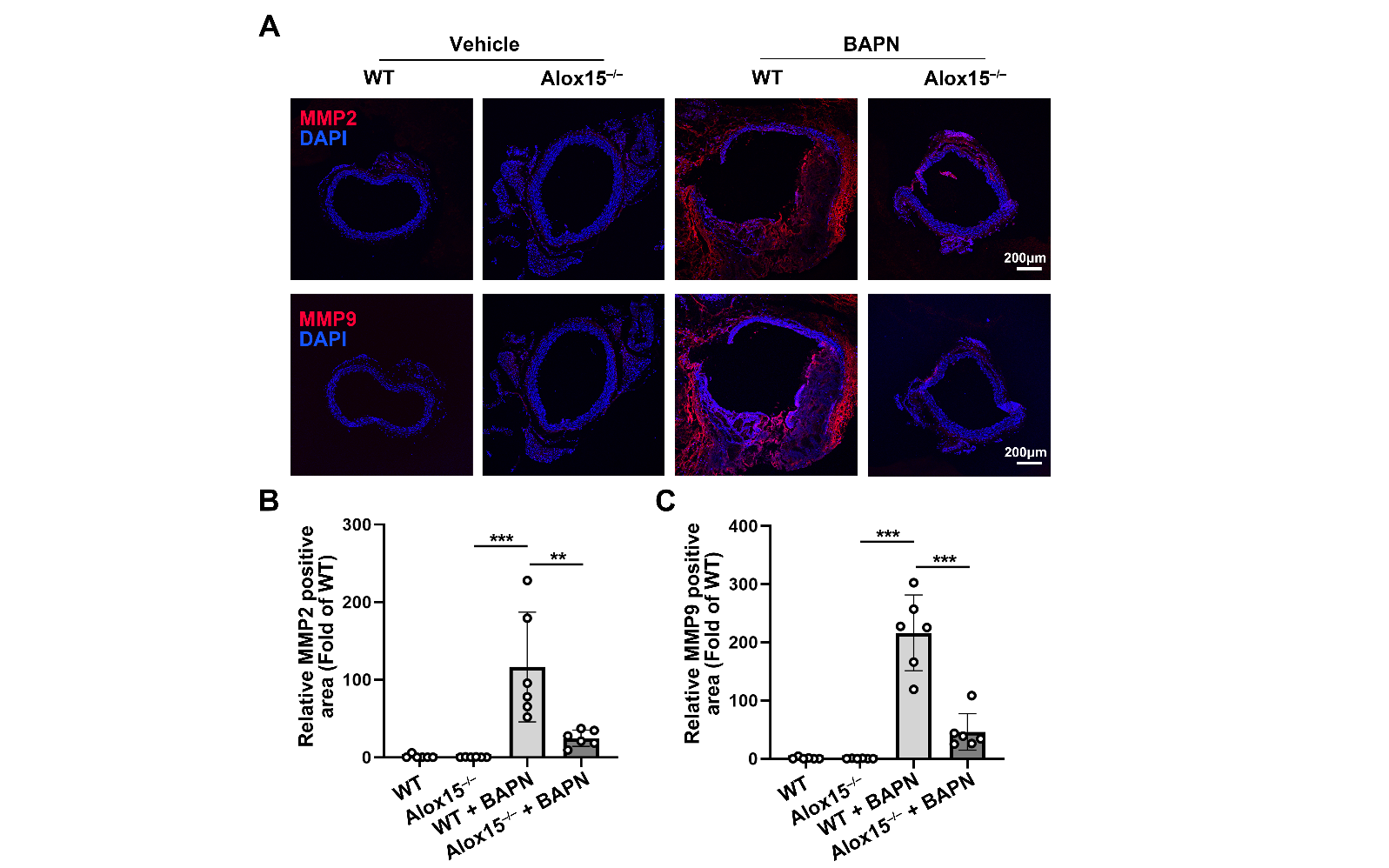


Figure S4**. A,** Immunofluorescence staining of MMP2 (Red) and MMP9 (Red) in aorta. Nuclei were counterstained with DAPI (blue; scale bars, 200 μm). **B** and **C,** Quantification of MMP2 and MMP9– positive area in aorta (n=6 per group). ***p* < 0.01 and ****p* < 0.001, data were presented as the mean±SD and analyzed by using one-way ANOVA, Tukey’s multiple comparisons test.


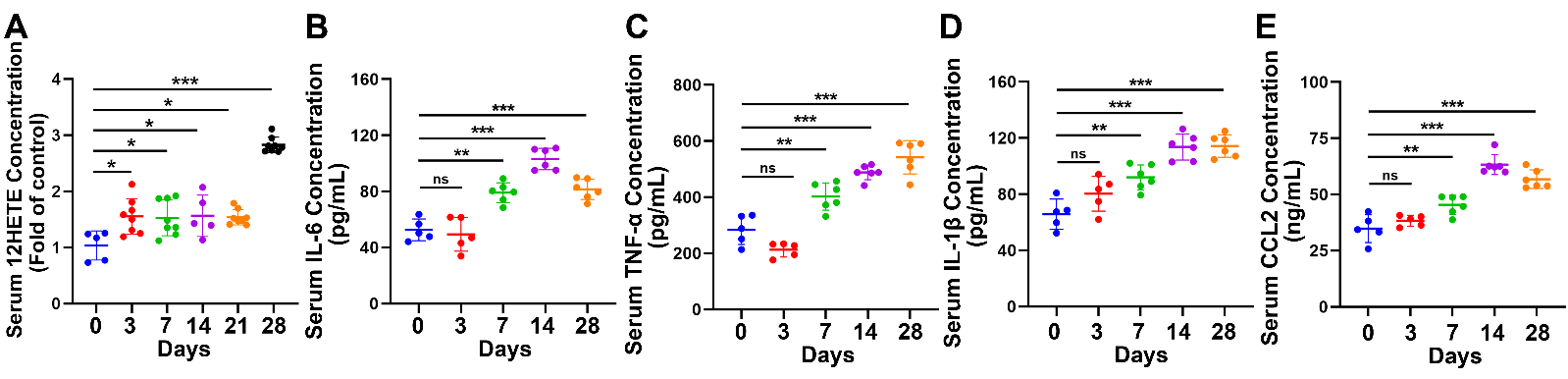


Figure S5**. A**, Relative concentrations of 12-HETE in BAPN-reduced TAD mice for 0, 3,7, 14, 21 and 28 days (n=5-8 per group). **B** – **E**, Relative IL-6, TNF-α, IL-1β and CCL2 concentrations in BAPN-reduced TAD mice for 0, 3,7, 14 and 28 days (n=5-8 per group). **p* < 0.05, ***p* < 0.01 and ****p* < 0.001. Data were presented as the mean±SD and analyzed by using one-way ANOVA, Tukey’s multiple comparisons test.


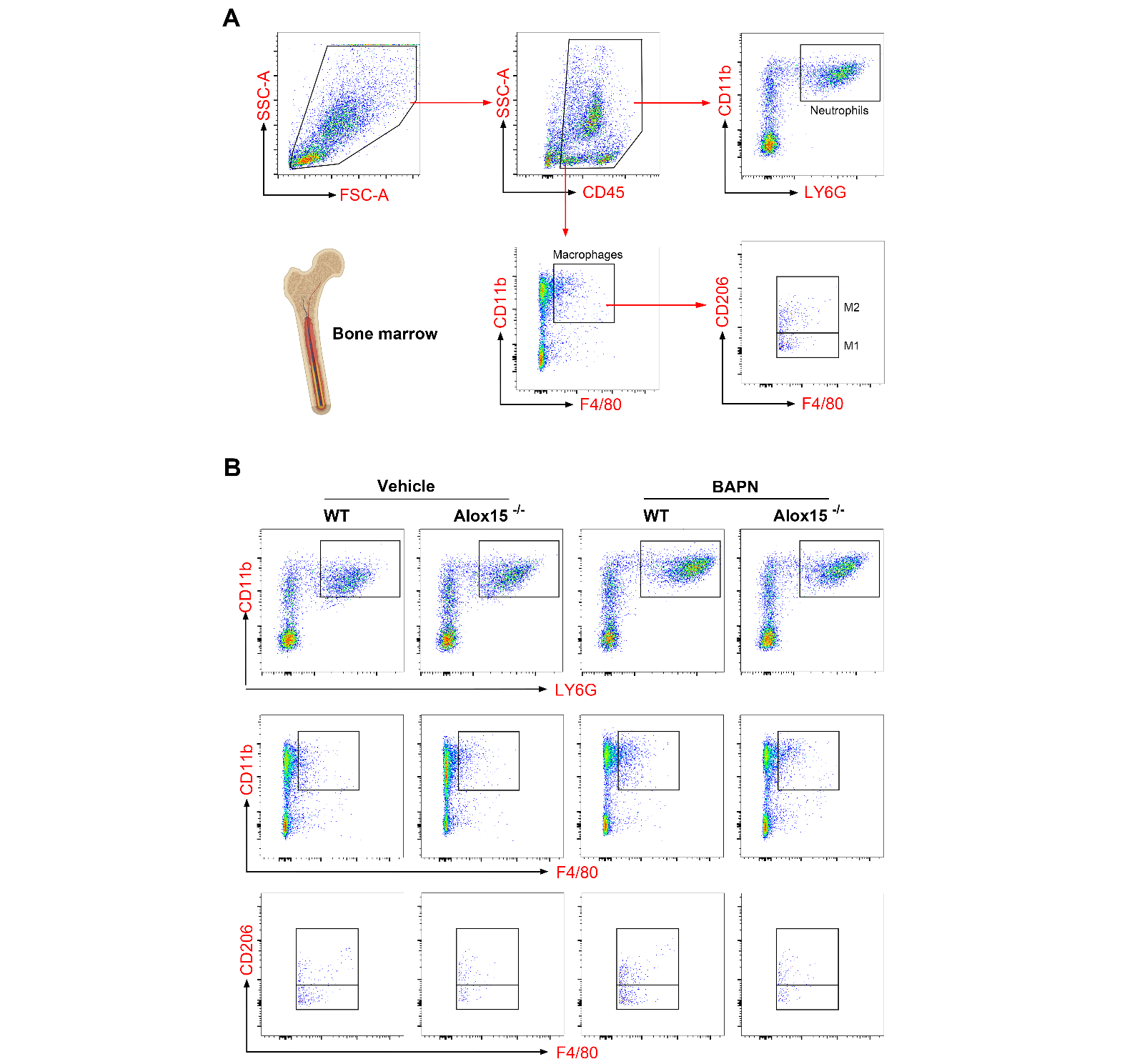


Figure S6**. A**, Gating strategy for identification of macrophages and neutrophils in mouse bone marrow. Macrophages were identified as CD45+CD11b+ F4/80+ and further classified as CD206+ and CD206-, neutrophils as CD45+CD11B+LY6G+. **B**, Representative flow cytometry analysis of bone marrow Ly6G+ CD11b+ neutrophils and CD11b + F4/80+ macrophages in BAPN - and saline-treated WT and Alox15^–/–^ mice.


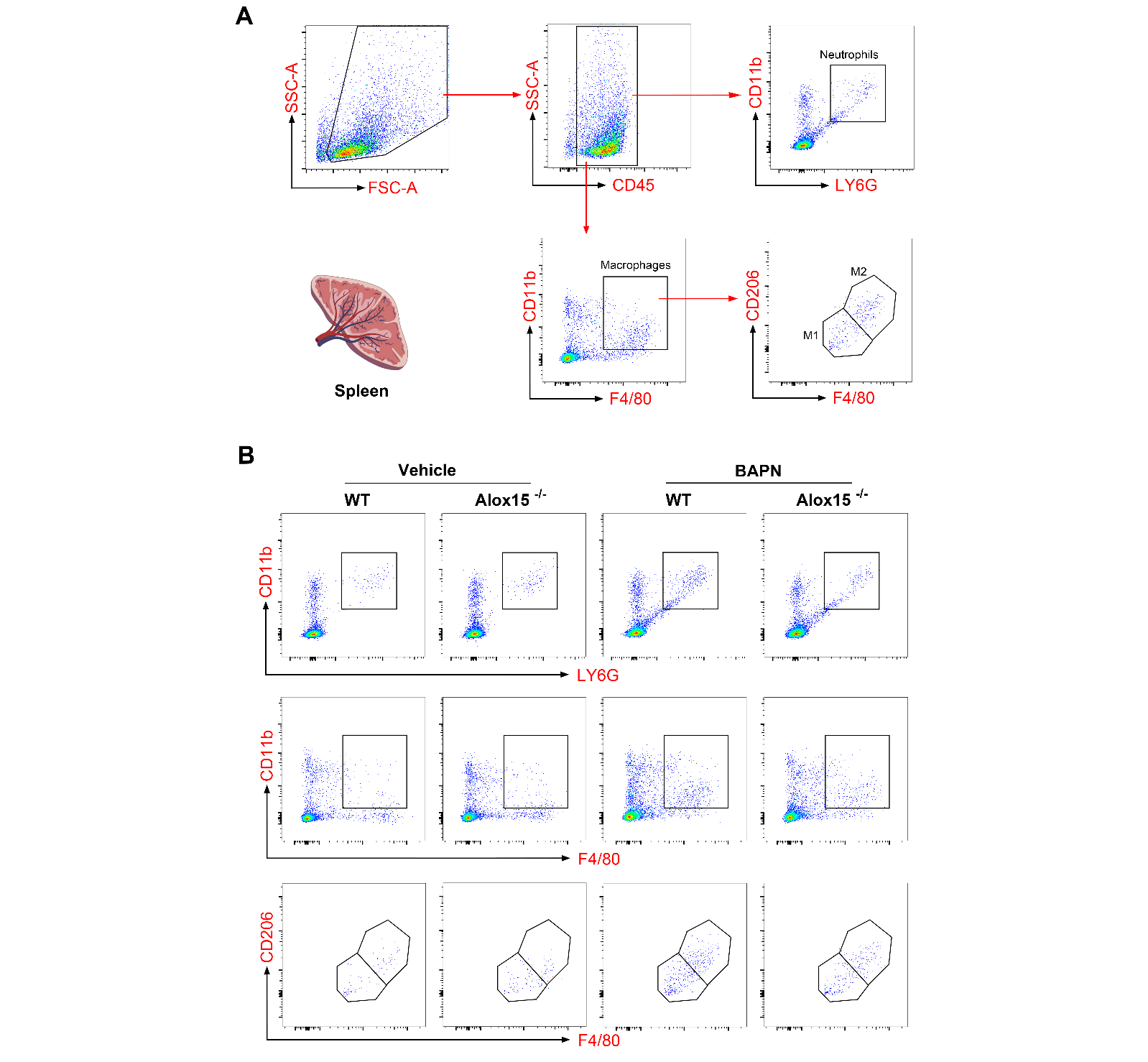


Figure S7**. A**, Gating strategy for identification of macrophages and neutrophils in mouse spleen. Macrophages were identified as CD45+CD11b+ F4/80+ and further classified as CD206+ and CD206-, neutrophils as CD45+CD11B+LY6G+. **B**, Representative flow cytometry analysis of spleen Ly6G+ CD11b+ neutrophils and CD11b + F4/80+ macrophages in BAPN - and saline-treated WT and Alox15^–/–^ mice.


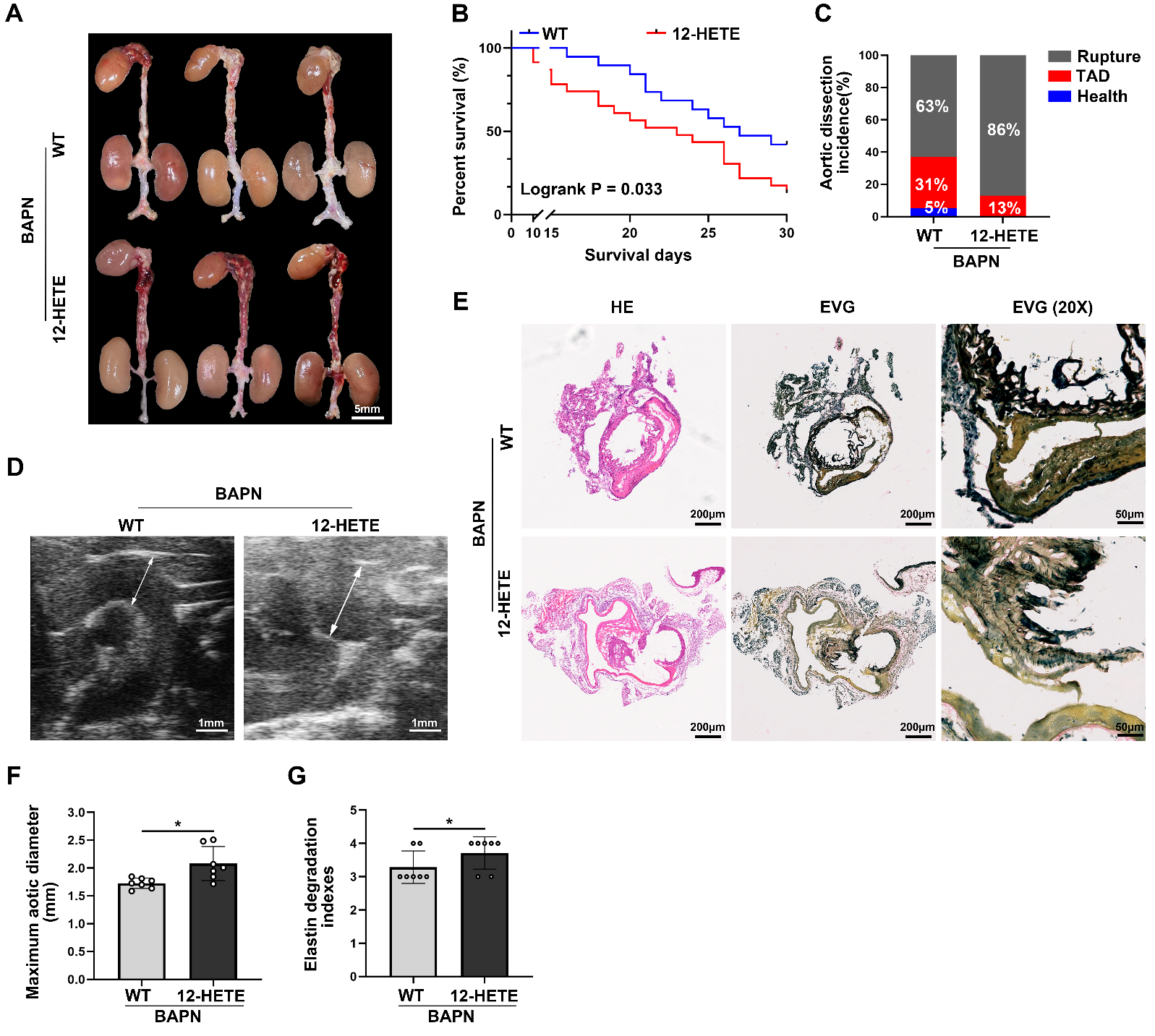


Figure S8**.** 12-HETE is an essential driver of TAD development via BLT2 receptor in mice. **A** through **G,** C57BL/6J mice were observed with or without 12-HETE after BAPN (β-aminopropionitrile monofumarate) treatment for 28 days. **A,** Representative macrographs of aorta (scale bar, 5 mm). **B,** Survival rate was estimated by Kaplan-Meier method and compared by log-rank test (n=19 and 23 per group). **C,** Thoracic aortic dissection (TAD) incidence (n=19 and 23 per group). **D,** Representative ultrasound images of thoracic aorta. **E,** Representative macroscopic images of aorta sections stained with hematoxylin and eosin (HE) and Elastic Van Gieson (EVG; scale bars, 200 μm and 50μm). **F,** Measurements of maximum aortic diameter (n=7 per group). **G,** Elastin break grades were analyzed by Kruskal-Wallis followed by Dunn multiple comparisons test (n=7 per group). **p* < 0.05. Data were presented as the mean±SD and analyzed by using an unpaired two-tailed Student’s t test.


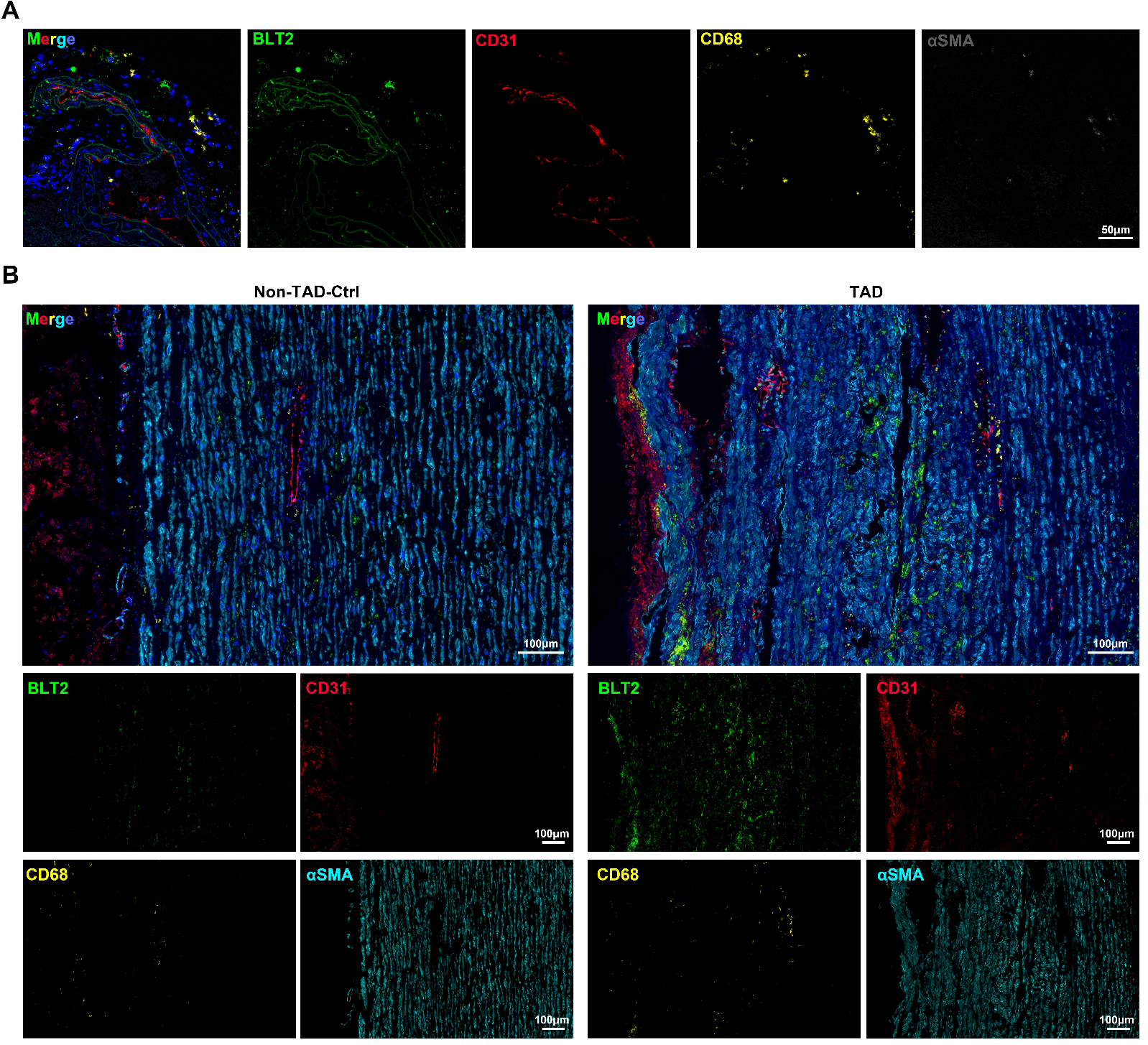


Figure S9**. A,** Representative confocal images of BLT2 (green) colocalized with CD68 (yellow)–positive macrophages, CD31 (red) positive endothelial cells and αSMA (Gray) positive smooth muscle cells in aorta tissues from TAD mouse. (scale bars, 50 μm). **B,** Representative confocal images of BLT2 (green) colocalized with CD68 (yellow)–positive macrophages, CD31 (red) positive endothelial cells and αSMA (cyan) positive smooth muscle cells in aorta tissues from non-TAD controls and TAD patients. (scale bars, 100 μm).


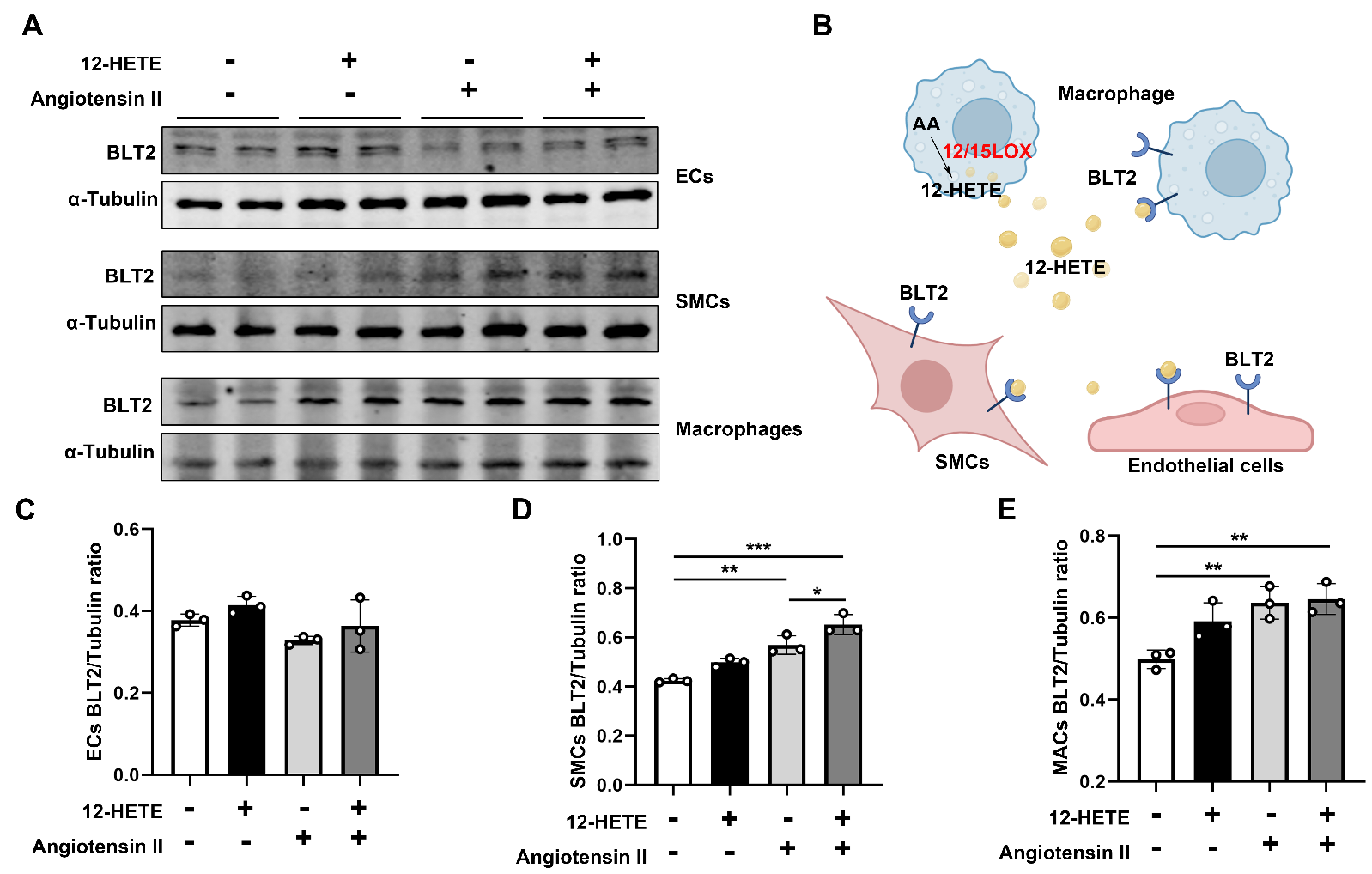


**Figure S10. A,** Representative Western blot images of BLT2 in MAECs, primary smooth muscle cells and primary macrophages. **B,** Graphical abstract. 12HETE combined with BLT2 expressed in macrophages, ECs and VSMCs. **C, D**, and **E,** Western blot analysis and quantification of BLT2 expressed in ECs, SMCs and macrophages. (n=3 per group).


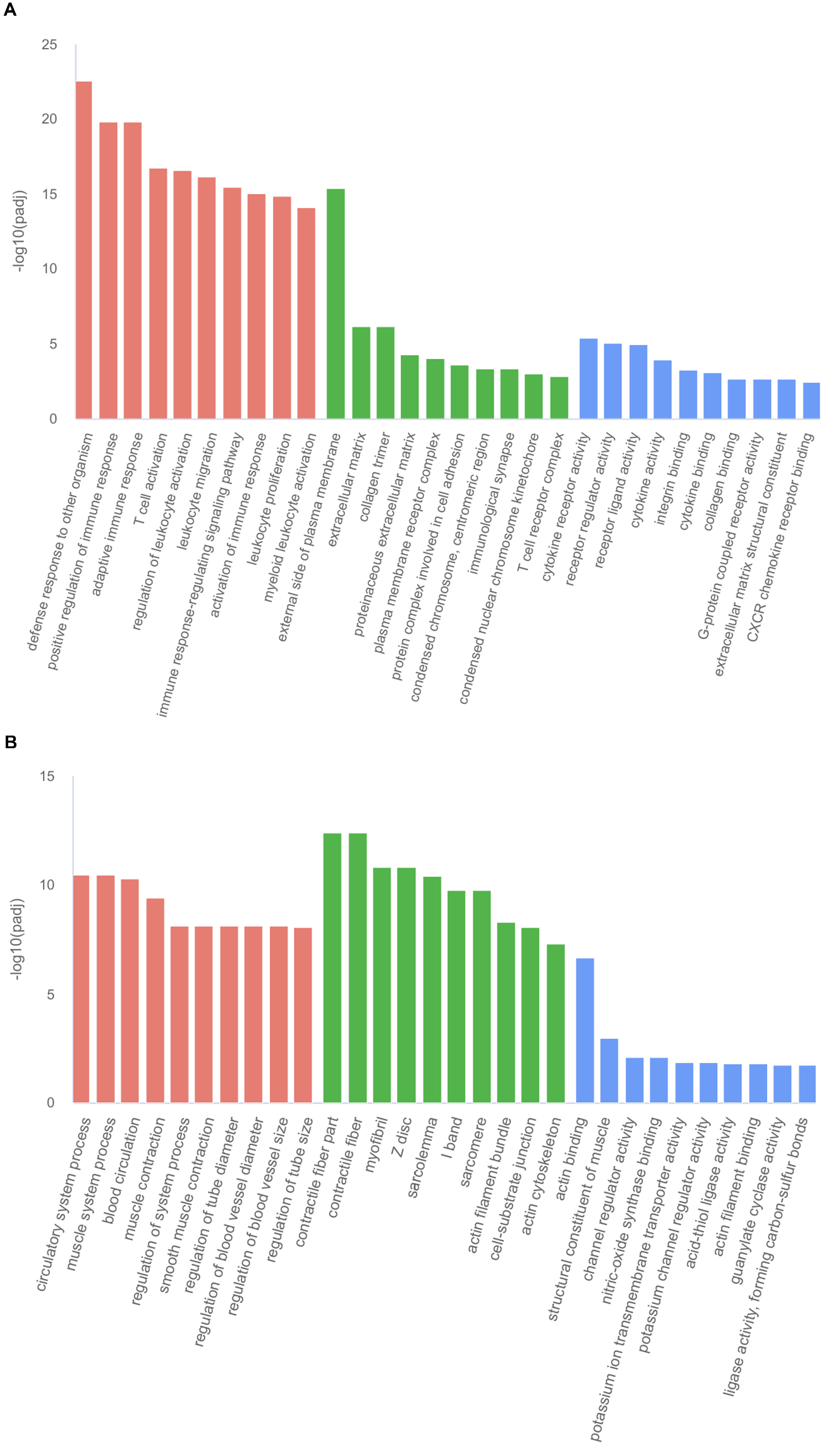


Figure S11. **A,** GO pathway enrichment analysis of the up-regulated genes in AAV-ALOX15 versus AAV-empty groups by R. **B,** GO pathway enrichment analysis of the down-regulated genes in AAV-ALOX15 versus AAV-empty groups by R.


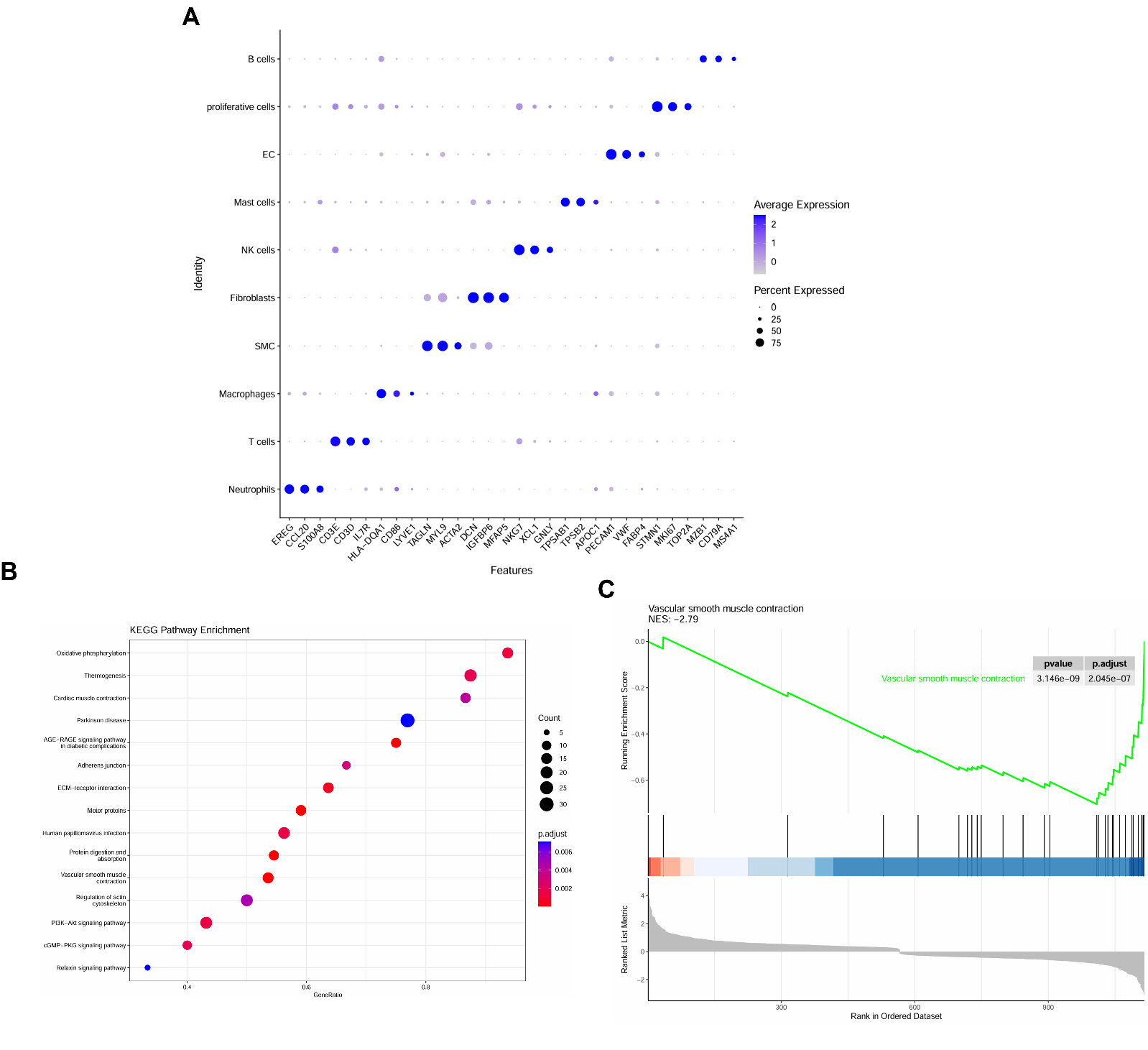


Figure S12**. A,** Expression of cell type markers used for cell type annotation. **B** and **C,** KEGG pathway analysis and GSEA analysis of smooth muscle contraction pathway of differential genes of SMC1 group in the control group and TAD group, the enrichment map of KEGG pathway. **C**, the enrichment fraction and statistical significance of smooth muscle contraction pathway.


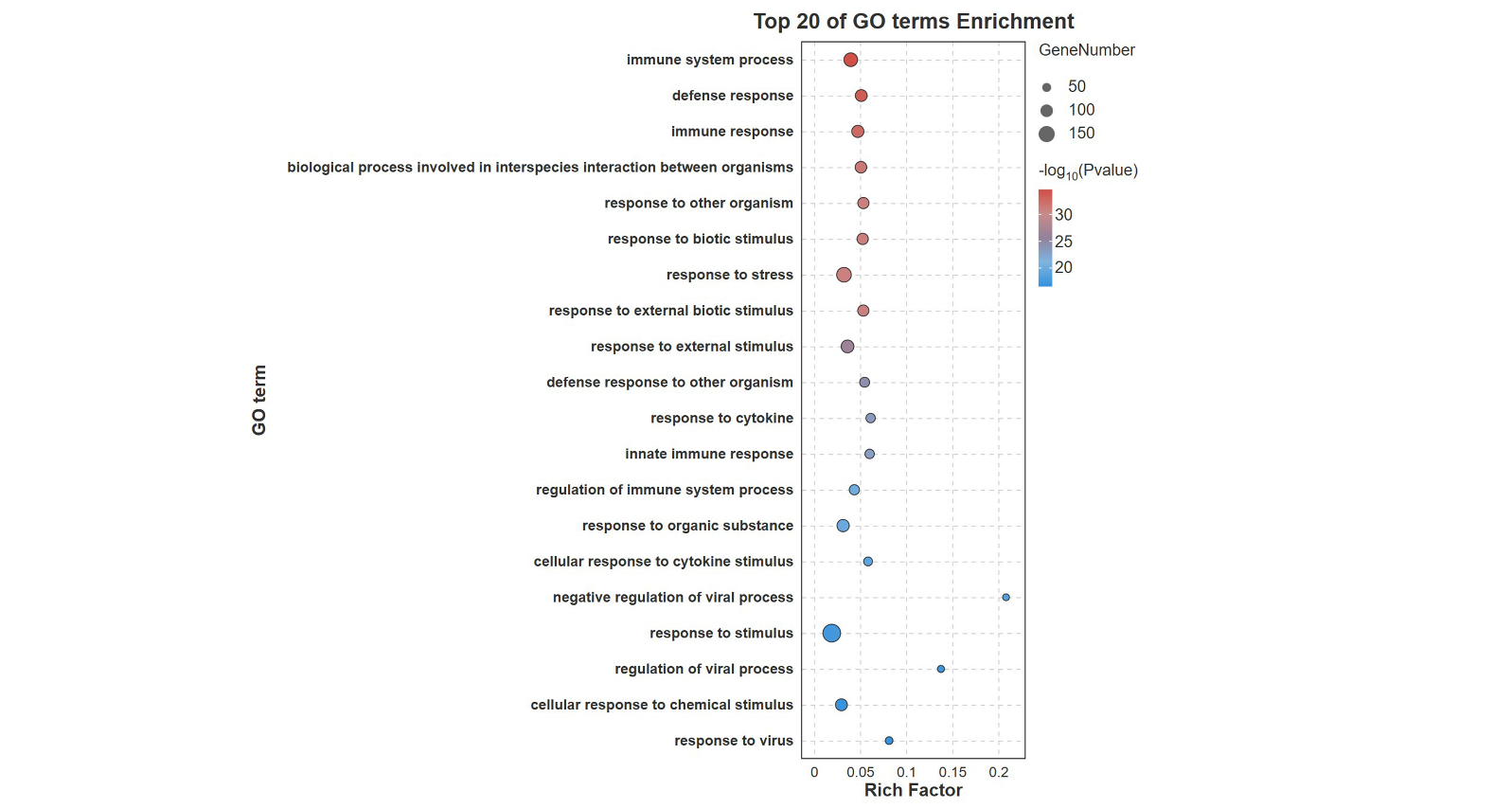


Figure S13. **A,** GO enrichment analysis of the myeloid cell cluster from human thoracic aortic dissection scRNA-seq data.


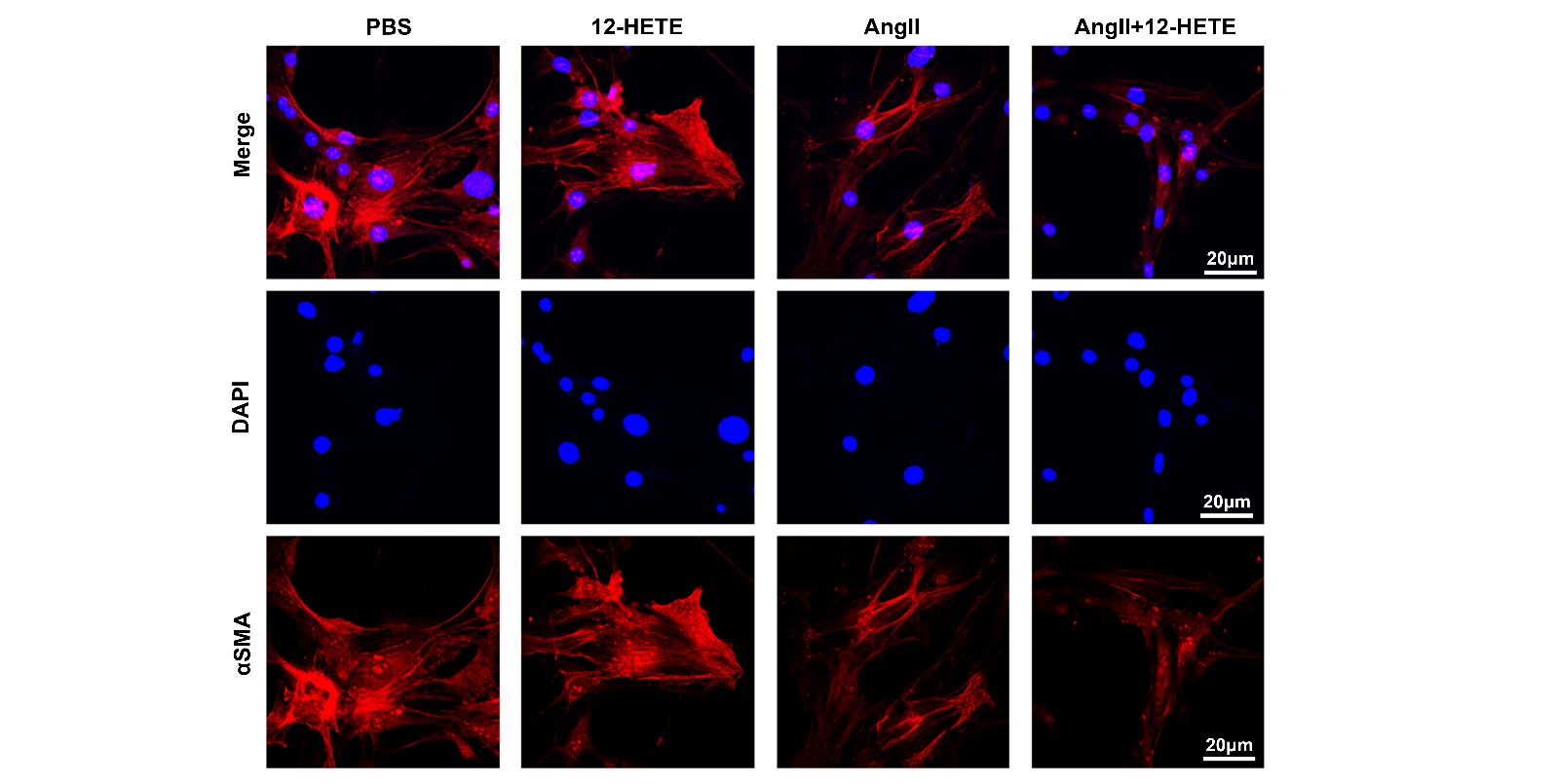


Figure S14**.** Representative confocal images of αSMA (red) in primary smooth muscle cells. (scale bar, 20 μm).


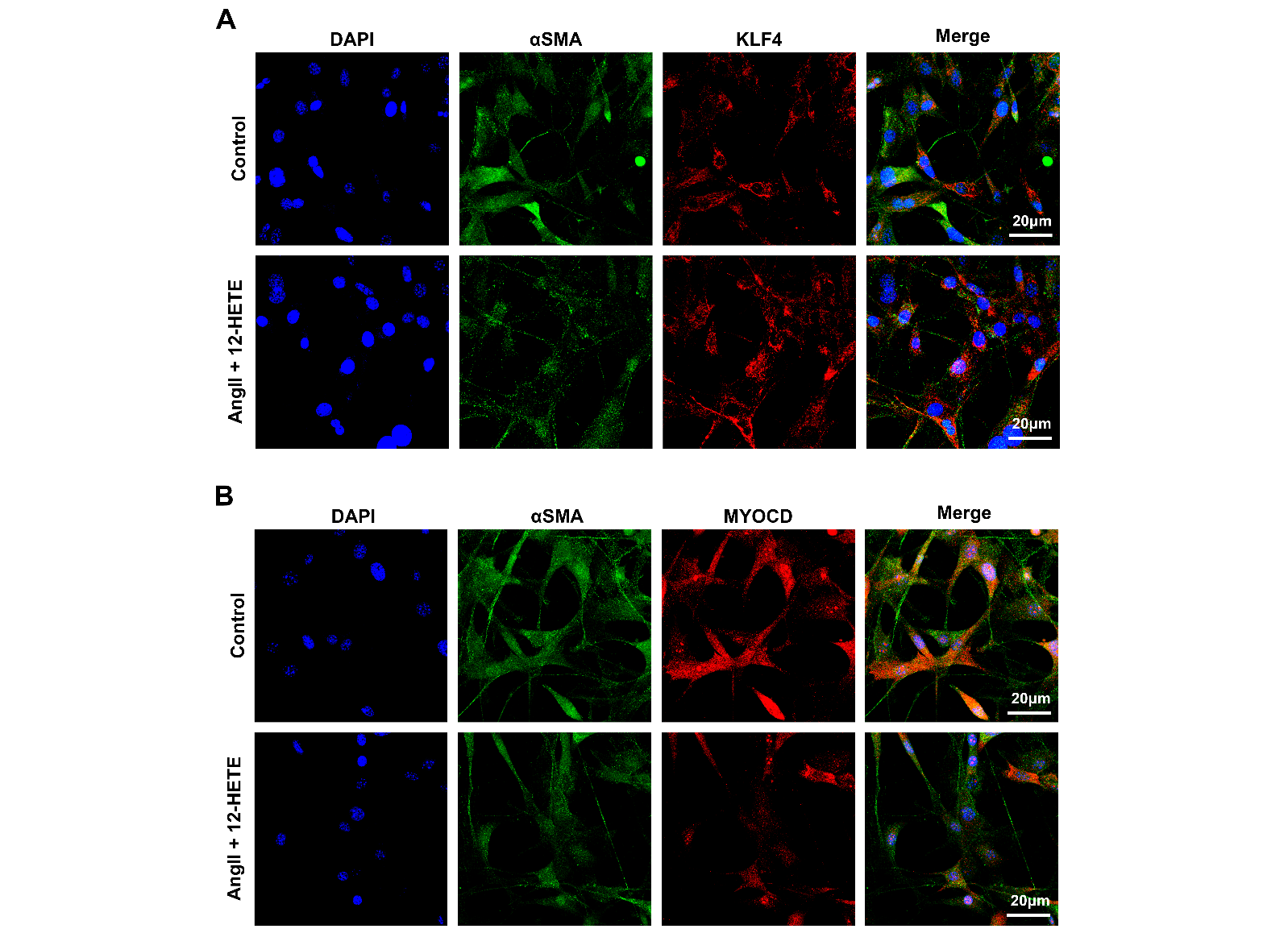


Figure S15**. A,** Representative confocal images of αSMA (green) and KLF4 (red) in primary smooth muscle cells. **B,** Representative confocal images of αSMA (green) and MYOCD (red) in primary smooth muscle cells. (scale bar, 20 μm).


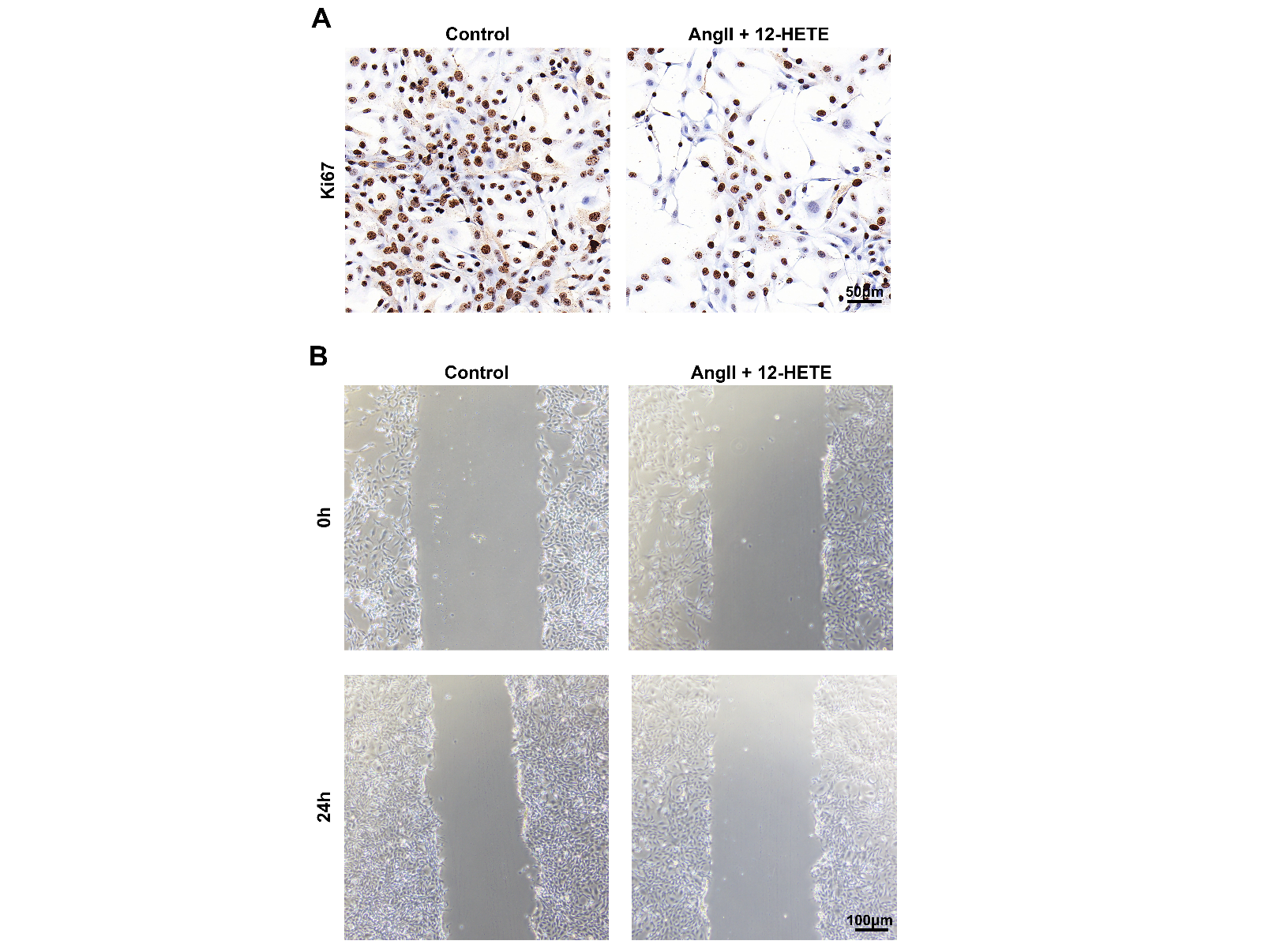


**Figure S16. A,** Representative images of immunofluorescence staining for the proliferation marker Ki67 in VSMCs under different treatment conditions (Control, Ang II+12-HETE). Nuclei are stained with DAPI (blue). Scale bar = 50 μm. **B,** Representative images from a wound-healing (scratch) assay demonstrating VSMC migration at 0 h and 24 h after treatment with PBS or Ang II+12-HETE. Scale bar = 100 μm.


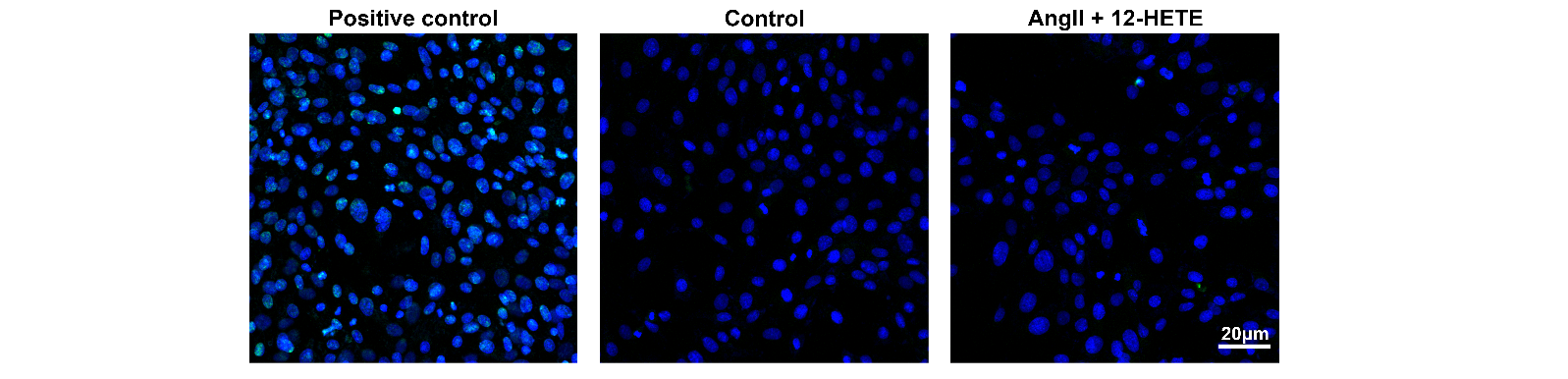


**Figure S17.** Representative fluorescence images of TUNEL staining in VSMCs under different treatment conditions (Positive Control, Ang II+12-HETE, and Control). Apoptotic nuclei are labeled in green (TUNEL), and all nuclei are counterstained with DAPI (blue). Scale bar = 20 μm.


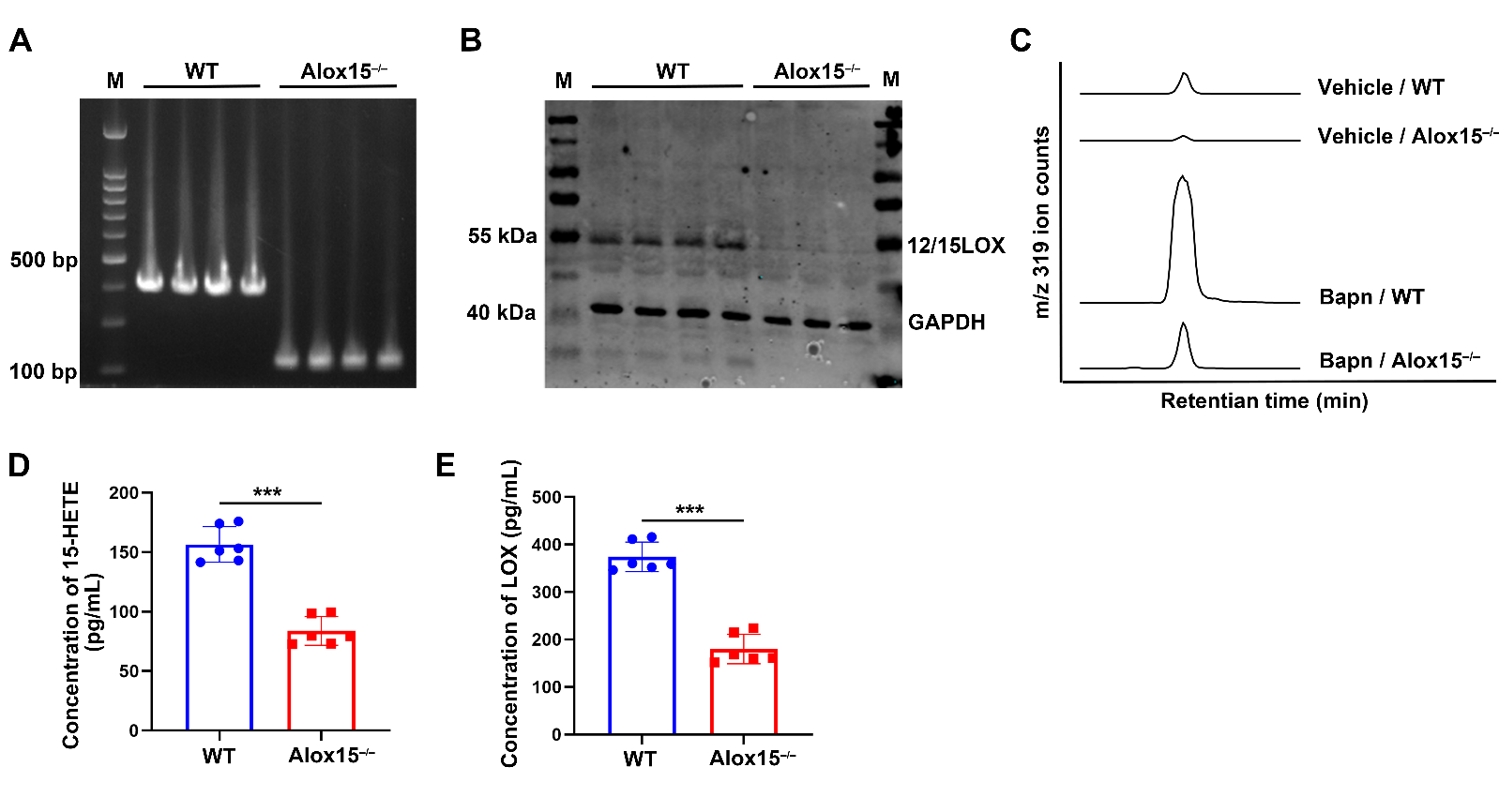


Figure S18**. A**, PCR analysis on genomic DNA derived from C57BL/6J and global Alox15 knockout mice. **B**, Western blot analysis of 12/15LOX expression in aortic tissues from C57BL/6J and global Alox15 knockout mice with BAPN inducement. **C**, Mass spectrometry analysis of 12-HETE expression in plasma from C57BL/6J and global Alox15 knockout mice with or without BAPN inducement. **D**, Relative 15HETE concentrations in WT and Alox15 knockout mice (n=6 per group). **E,** Relative LOX concentrations in WT and Alox15 knockout mice (n=6 per group). ****p* < 0.001. Data were presented as the mean±SD and analyzed by using an unpaired two-tailed Student’s t test.

# SUPPLEMETARY TABLES

## Supplemental Table I. Baseline characteristics of AD patients and controls.

|  | Control | Aortic disection | | | p value (TAAD vs TBAD) |
| --- | --- | --- | --- | --- | --- |
|  |  | All patients | Stanford type A | Stanford type B |  |
| N | 22 | 22 | 12 | 10 |  |
| Clinical Characteristics |  |  |  |  |  |
| Age, median (IQR), year | 44.7±11.2 | 47.0±12.5 | 48.9±12.9 | 44.7±12.3 | 0.444 |
| Male gender, *n* (%) | 13 (59.1%) | 13 (59.1%) | 6 (50.0%) | 7 (70.0%) | 0.415 |
| SBP, median (IQR), mmHg | 110±11.5 | 131±22.6 | 128±24.2 | 133±21.3 | 0.62 |
| DBP, median (IQR), mmHg | 68.9±8.44 | 72.4±12.4 | 73.6±12.8 | 70.8±12.5 | 0.62 |
| Heart rate, median (IQR), beats/min | 68.5±7.29 | 83.0±14.2 | 82.6±10.6 | 83.6±18.7 | 0.748 |
| Risk factors |  |  |  |  |  |
| Smoking, *n* (%) | 4 (18.2%) | 6 (28.6%) | 3 (25.0%) | 3 (33.3%) | 1 |
| Alcohol use, *n* (%) | 4 (18.2%) | 4 (19.0%) | 2 (16.7%) | 2 (22.2%) | 1 |
| Hypertension, *n* (%) | 5 (22.7%) | 16 (72.7%) | 10 (83.3%) | 6 (60.0%) | 0.348 |
| Diabetes, *n* (%) | 2 (9.09%) | 1 (4.55%) | 1 (8.33%) | 0 (0.00%) | 1 |
| BMI, mean±SD,kg/m2 | 22.6±2.52 | 25.4±4.44 | 24.9±3.88 | 26.1±5.17 | 0.549 |
| Laboratory Examination |  |  |  |  |  |
| Glucose, median (IQR), mmol/L | 5.11±0.69 | 6.95±2.46 | 6.20±2.02 | 7.85±2.73 | 0.132 |
| LDL-c, median (IQR), mmol/L | 2.76±0.77 | 2.74±0.58 | 2.66±0.56 | 2.88±0.68 | 0.608 |
| HDL-c, median (IQR), mmol/L | 1.30±0.26 | 1.01±0.21 | 1.07±0.17 | 0.91±0.27 | 0.329 |
| TG, median (IQR), mmol/L | 1.03±0.57 | 0.99±0.35 | 0.99±0.36 | 0.99±0.38 | 0.995 |
| TC, median (IQR), mmol/L | 4.56±0.94 | 4.25±0.63 | 4.29±0.69 | 4.20±0.59 | 0.827 |
| Creatinine, median (IQR), μmol/L | 72.8±11.3 | 79.7±30.0 | 87.8±37.1 | 70.0±14.7 | 0.148 |

* Major adverse events：occurrence of acute heart failure, renal failure, respiratory distress syndrome, neurological ischemic damage, septicemia, and/or the need for thoracotomy. Data are presented as n (%) for categorical data, medians (IQRs) for continuous data. P values are for comparisons between controls and patients (chi-square test was used for categorical variables and Wilcoxon test was used for continuous variables). P≤0.05 was considered statistically significant.

## Supplemental Table II. Baseline characteristics of AD patients and controls in the single-center validation cohort.

|  | Control | Aortic disection | p value |
| --- | --- | --- | --- |
| N | 138 | 218 |  |
| **Clinical Characteristics** |  |  |  |
| Age, mean ± SD, year | 49.7±11.6 | 48.7±10.6 | 0.426 |
| Male gender, *n* (%) | 82 (59.4%) | 175 (80.3%) | <0.001 |
| SBP, mean ± SD, mmHg | 124±18.1 | 127±19.2 | 0.09 |
| DBP, mean ± SD, mmHg | 74.2±13.9 | 70.3±12.3 | 0.008 |
| **Risk factors** |  |  |  |
| Smoking, *n* (%) | 41 (29.7%) | 103 (47.2%) | 0.002 |
| Alcohol use, *n* (%) | 35 (25.4%) | 10 (4.59%) | <0.001 |
| Hypertension, *n* (%) | 51 (37.0%) | 149 (68.3%) | <0.001 |
| Diabetes, *n* (%) | 25 (18.1%) | 9 (4.13%) | <0.001 |
| BMI, mean ± SD, kg/m2 | 24.6±3.51 | 26.2±3.77 | <0.001 |
| **Laboratory Examination** |  |  |  |
| Glucose, mean ± SD, mmol/L | 5.68±1.55 | 5.54±1.00 | 0.357 |
| TC, mean ± SD, mmol/L | 5.11±0.96 | 4.62±0.65 | <0.001 |
| TG, mean ± SD, mmol/L | 1.86±2.30 | 1.49±0.84 | 0.071 |
| 12-HETE, ng/mL | 4.01±2.44 | 20.7±25.5 | <0.001 |

## Supplemental Table III. Primers for real-time PCR analysis in human and mice.

| homo -ALOX15 F | GGGCAAGGAGACAGAACTCAA |
| --- | --- |
| homo -ALOX15 R | CAGCGGTAACAAGGGAACCT |
| homo-β-actin F | CATGTACGTTGCTATCCAGGC |
| homo-β-actin R | CTCCTTAATGTCACGCACGAT |
| Mouse-ALOX15 F | GCTGCCCAATCCTAATCAGTC |
| Mouse-ALOX15 R | TTCCTTATCCAAGGCAGCCAG |
| Mouse-GADPH F | GGGTCCCAGCTTAGGTTCATC |
| Mouse-GADPH R | AATCCGTTCACACCGACCTT |

## Supplemental Table IV. Antibodies.

| Antibody | Vendor name | Catalog number | Dilution | Application |
| --- | --- | --- | --- | --- |
| 12/15LOX | Santa Cruz | sc-133085 | 1:1000; 1:500 | WB, IF |
| 12/15LOX | Bioss | bs-6505R | 1:500 | IF |
| CD68 | Santa Cruz | sc-20060 | 1:500 | IF |
| Collagen I | Abclonal | A16891 | 1:1000; 1:500 | WB, IF |
| α-SMA | CST | 48938S | 1:1000; 1:500 | WB, IF |
| SM22α | Santa Cruz | sc-53932 | 1:1000; 1:500 | WB, IF |
| α-Tubulin | CST | 2144S | 1:1000 | WB |
| GAPDH | CST | 2118S | 1:1000 | WB |
| LY6G | Abclonal | A20861 | 1:500 | IF |
| CD45 | Biolegend | 103128 | 1:200 | FC |
| CD11b | Biolegend | 101257 | 1:200 | FC |
| LY6G | Biolegend | 127616 | 1:200 | FC |
| F4/80 | Biolegend | 123137 | 1:200 | FC |
| CD206 | BDPMG | 565250 | 1:200 | FC |
| BLT2 | Cayman | 120124 | 1:1000 | WB |
| CD31 | Santa Cruz | sc-376734 | 1:500 | IF |
| NOX1 | Proteintech | 17772-1-AP | 1:1000 | WB |
| p-IκBb | Santa Cruz | sc-8404 | 1:1000 | WB |
| IκBb | Santa Cruz | sc-1643 | 1:1000 | WB |
| IL6 | Santa Cruz | sc-35596 | 1:1000 | WB |
| p-JAK | Abclonal | AP0531 | 1:1000 | WB |
| JAK | Santa Cruz | sc-390539 | 1:1000 | WB |
| p-STAT | CST | 9145S | 1:1000 | WB |
| STAT | CST | 9139S | 1:1000 | WB |
| MMP2 | Santa Cruz | sc-13594 | 1:500 | IF |
| MMP9 | Santa Cruz | sc-393859 | 1:500 | IF |

**Supplemental Table V. Significantly changed metabolites in human plasma.**

| Metabolite | FC | log2(FC) | FDR | VIP |
| --- | --- | --- | --- | --- |
| 12-HETE | 6.5869 | 2.7196 | 4.66E-14 | 2.550654 |
| 8-HETE | 6.9709 | 2.8014 | 4.66E-14 | 2.5307 |
| HETE | 2.9746 | 1.5727 | 2.34E-09 | 2.365324 |
| 12-HEPE | 6.5395 | 2.7092 | 3.01E-09 | 2.32603 |
| 10-HDHA | 3.2097 | 1.6825 | 6.25E-06 | 1.953214 |
| 8-HEPE | 2.8867 | 1.5294 | 6.25E-06 | 1.999144 |
| 14-HDHA | 3.0037 | 1.5867 | 1.67E-05 | 1.908261 |
| HEPE | 2.5157 | 1.331 | 3.26E-05 | 1.871307 |
| TXB2 | 5.9521 | 2.5734 | 0.002079 | 1.625226 |
| HDHA | 2.1132 | 1.0794 | 0.002085 | 1.556404 |
| 8-HDHA | 0.61711 | -0.6964 | 0.007489 | 1.441354 |
| FA22:4 | 1.7639 | 0.81875 | 0.01543 | 1.001038 |
| PGE3 | 0.14155 | -2.8206 | 0.024895 | 1.164353 |
| 9-HODE | 0.80968 | -0.30457 | 0.16157 | 1.03616 |
| FA22:6 | 0.82425 | -0.27884 | 0.19932 | 0.952627 |
| FA20:3 | 1.3621 | 0.44582 | 0.26435 | 0.57994 |
| 12,13-diHOME | 0.86494 | -0.20932 | 0.28718 | 0.838877 |
| 19-HETE | 1.1404 | 0.18958 | 0.29124 | 0.723221 |
| FA20:5 | 1.4416 | 0.52763 | 0.31377 | 0.60581 |
| 17-HDHA | 0.89193 | -0.165 | 0.31377 | 0.762585 |
| HODE | 0.88482 | -0.17655 | 0.31816 | 0.846712 |
| FA22:5 | 1.3061 | 0.38524 | 0.3659 | 0.498542 |
| 5-HEPE | 1.3735 | 0.45783 | 0.39317 | 0.576694 |
| 4-HDHA | 1.3615 | 0.44515 | 0.39626 | 0.635252 |
| 7-HDHA | 0.89902 | -0.15358 | 0.39626 | 0.38197 |
| 5-HETE | 1.3163 | 0.3965 | 0.42553 | 0.645336 |
| diHOME | 0.79864 | -0.32438 | 0.43441 | 0.706917 |
| FA20:4 | 1.1592 | 0.21308 | 0.43441 | 0.371109 |
| 13-HODE | 0.95907 | -0.06029 | 0.53634 | 0.653138 |
| PGF2a | 1.3066 | 0.38579 | 0.54014 | 0.500004 |
| FA18:3.g | 0.90313 | -0.14699 | 0.62398 | 0.507817 |
| PGD2 | 0.80525 | -0.3125 | 0.62398 | 0.335297 |
| 11b-PGF2a | 0.67471 | -0.56765 | 0.62398 | 0.385768 |
| 9,10-.diHOME | 0.76149 | -0.39311 | 0.62398 | 0.590996 |
| FA18:2 | 0.93634 | -0.0949 | 0.63692 | 0.584453 |
| oxo-ETE | 1.373 | 0.45731 | 0.64945 | 0.307349 |
| PG metabolite | 1.3837 | 0.46853 | 0.64945 | 0.383908 |
| 15-HETE | 0.968 | -0.04692 | 0.64945 | 0.198481 |
| 15-oxo-ETE | 1.4013 | 0.48673 | 0.64945 | 0.310732 |
| 6k-PGF1a | 1.4317 | 0.51774 | 0.69031 | 0.263142 |
| DHET | 1.3674 | 0.45141 | 0.75379 | 0.353087 |
| PGF2b | 0.79689 | -0.32754 | 0.75379 | 0.31909 |
| 9-HEPE | 1.2399 | 0.31022 | 0.79169 | 0.136918 |
| PGB3 | 0.87588 | -0.19119 | 0.79437 | 0.200782 |
| 5,6-DHET | 1.802 | 0.84959 | 0.81695 | 0.395353 |
| FA18:4 | 0.98054 | -0.02835 | 0.81742 | 0.376678 |
| Free fatty acids | 1.0427 | 0.060311 | 0.82323 | 0.053309 |
| 18-HEPE | 1.1566 | 0.20985 | 0.82323 | 0.1387 |
| 5-oxo-ETE | 1.0696 | 0.097131 | 0.82323 | 0.29288 |
| 8,9-DHET | 1.2188 | 0.28547 | 0.83708 | 0.181809 |
| PG | 0.89945 | -0.15289 | 0.92454 | 0.030215 |
| DHA.metabolites | 1.6461 | 0.71906 | 0.97863 | 0.127544 |
| EDP | 1.4691 | 0.55491 | 0.97863 | 0.122195 |
| EET | 1.4085 | 0.49411 | 0.97863 | 0.146297 |
| FA18:3a | 1.1209 | 0.16468 | 0.97863 | 0.207109 |
| resolvin.D1 | 1.6461 | 0.71906 | 0.97863 | 0.127544 |
| 11,12-EET | 1.4317 | 0.51777 | 0.97863 | 0.179527 |
| 16,17-EDP | 1.4691 | 0.55491 | 0.97863 | 0.122195 |
| 8,9-.EET | 1.3963 | 0.48156 | 0.97863 | 0.135504 |

**Supplemental Table Ⅵ. Significantly changed metabolites in mice plasma.**

| Metabolite | FC | log2(FC) | FDR | VIP |
| --- | --- | --- | --- | --- |
| ARA | 1434.9 | 10.487 | 2.95E-07 | 1.6292 |
| DHA | 13.322 | 3.7357 | 0.000104 | 1.3288 |
| DPA | 9.2245 | 3.2055 | 1.91E-06 | 1.5462 |
| EPA | 3.0399 | 1.604 | 0.001259 | 1.1511 |
| LTB4 | 2.4914 | 1.317 | 0.009322 | 0.97601 |
| LXA4 | 0.37162 | -1.4281 | 0.05745 | 0.73555 |
| maresin.1 | 0.27674 | -1.8534 | 0.012758 | 0.93917 |
| PGB2 | 1347.2 | 10.396 | 2.57E-05 | 1.4174 |
| PGB3 | 0.95082 | -0.07276 | 0.66698 | 0.22248 |
| PGD2 | 42.361 | 5.4047 | 5.55E-05 | 1.3703 |
| PGD3 | 3.5429 | 1.8249 | 0.084902 | 0.68059 |
| PGE2 | 7.9453 | 2.9901 | 2.35E-05 | 1.4255 |
| PGE3 | 1.855 | 0.89145 | 0.1987 | 0.54927 |
| PGF2a | 202.69 | 7.6632 | 5.13E-11 | 1.8683 |
| PGF3a | 1.2031 | 0.26675 | 0.83527 | 0.10825 |
| PGJ2 | 1319.7 | 10.366 | 3.80E-06 | 1.5136 |
| resolvin.D1 | 2.5089 | 1.3271 | 0.38849 | 0.38813 |
| resolvin.D2 | 1.0687 | 0.095818 | 0.94862 | 0.028105 |
| TXB2 | 39.131 | 5.2903 | 1.01E-11 | 1.9035 |
| TXB3 | 14.253 | 3.8331 | 4.01E-07 | 1.614 |
| X10.HDoHE | 0.91917 | -0.12159 | 0.2039 | 0.54098 |
| X10.s..17.s..DiHOME | 0.4074 | -1.2955 | 0.57647 | 0.27993 |
| X11.12.EET | 4.7206 | 2.239 | 0.012047 | 0.94824 |
| X11.HDoHE | 0.83097 | -0.26713 | 0.80506 | 0.14562 |
| X11.HEPE | 0.76224 | -0.39169 | 0.62184 | 0.2518 |
| X11.HETE | 1427.4 | 10.479 | 1.09E-12 | 1.9608 |
| X11_12.DHET | 2.1603 | 1.1112 | 0.020558 | 0.87151 |
| X11_12.EEQ | 37.048 | 5.2113 | 0.000111 | 1.3218 |
| X12.13.DiHOME | 0.72146 | -0.471 | 0.94511 | 0.040853 |
| X12.13.EpOME | 1.0087 | 0.012531 | 0.80506 | 0.13099 |
| X12.HEPE | 1.9752 | 0.98202 | 0.002397 | 1.0988 |
| X12.HETE | 1434.4 | 10.486 | 1.09E-12 | 1.9605 |
| X13.HDoHE | 1.6407 | 0.71431 | 0.020558 | 0.87168 |
| X14.HDoHE | 0.87804 | -0.18763 | 0.25084 | 0.49727 |
| X14_15.DHET | 3.1798 | 1.6689 | 0.003492 | 1.0652 |
| X14_15.DiHETE | 0.61622 | -0.69847 | 0.54802 | 0.2971 |
| X14_15.EEQ | 1.2416 | 0.3122 | 0.38193 | 0.26154 |
| X14_15.EET | 2.0523 | 1.0373 | 0.05745 | 0.73786 |
| X15.deoxy.PGJ2 | 0.86674 | -0.20633 | 0.66698 | 0.223 |
| X15.HEPE | 0.78237 | -0.35407 | 0.98579 | 0.006217 |
| X15.HETE | 1449.9 | 10.502 | 1.47E-12 | 1.9493 |
| X15.oxo.ETE | 0.68058 | -0.55517 | 0.73108 | 0.18632 |
| X16.HDoHE | 1.27 | 0.34488 | 0.22919 | 0.51713 |
| X16.HETE | 2.1174 | 1.0823 | 0.04607 | 0.76958 |
| X16_17.EDP | 0.71971 | -0.47451 | 0.83527 | 0.11174 |
| X17.HDoHE | 0.83696 | -0.25677 | 0.3648 | 0.40959 |
| X17.HETE | 2.3377 | 1.2251 | 0.022327 | 0.85895 |
| X17_18.DiHETE | 0.62477 | -0.67861 | 0.43773 | 0.35816 |
| X17_18.EEQ | 0.6528 | -0.61528 | 0.80506 | 0.13123 |
| X18.HEPE | 0.73008 | -0.45388 | 0.88378 | 0.082913 |
| X18.HETE | 2.7267 | 1.4472 | 0.043733 | 0.77918 |
| X19.HETE | 7.3347 | 2.8747 | 1.91E-06 | 1.5469 |
| X19_20.EDP | 2.7022 | 1.4341 | 0.01839 | 0.89229 |
| X20.HDoHE | 0.76448 | -0.38745 | 0.62184 | 0.25101 |
| X20.HETE | 6.389 | 2.6756 | 0.013197 | 0.93244 |
| X4.HDoHE | 2.735 | 1.4515 | 0.000223 | 1.274 |
| X5.HEPE | 1.2789 | 0.35491 | 0.32145 | 0.44731 |
| X5.HETE | 2.6515 | 1.4068 | 0.000281 | 1.2566 |
| X5.oxo.ETE | 0.40522 | -1.3032 | 0.35848 | 0.41737 |
| X5_6.DHET | 3.6806 | 1.8799 | 9.79E-05 | 1.3355 |
| X5_6.diHETE | 0.81594 | -0.29347 | 0.54802 | 0.30099 |
| X5_6.EET | 0.52126 | -0.93993 | 0.94862 | 0.029269 |
| X6.keto.PGF1a | 3.324 | 1.7329 | 0.013214 | 0.92898 |
| X7.HDoHE | 0.55995 | -0.83664 | 0.89327 | 0.068212 |
| X8.HDoHE | 0.80677 | -0.30977 | 0.75376 | 0.17212 |
| X8.HEPE | 0.7459 | -0.42294 | 0.72891 | 0.19217 |
| X8.HETE | 1506.4 | 10.557 | 1.89E-12 | 1.9366 |
| X8_9.DHET | 1.5046 | 0.58942 | 0.35848 | 0.42053 |
| X8_9.EEQ | 1324.6 | 10.371 | 0.000168 | 1.2942 |
| X8_9.EET | 1444.6 | 10.496 | 5.22E-05 | 1.377 |
| X9.10.DiHOME | 0.76273 | -0.39075 | 0.80506 | 0.13471 |
| X9.10.EpOME | 0.91887 | -0.12207 | 0.89327 | 0.070566 |
| X9.HEPE | 1.4944 | 0.57959 | 0.020558 | 0.87091 |
| X9.HETE | 1517.1 | 10.567 | 1.89E-12 | 1.9379 |
